# The Trade-offs between Wildfires and Prescribed Fires: A Case Study for 2016 Gatlinburg Wildfires

**DOI:** 10.1021/acsestair.4c00233

**Published:** 2025-01-09

**Authors:** Zongrun Li, Ambarish Vaidyanathan, Kamal J. Maji, Yongtao Hu, Susan M. O’Neill, Armistead G. Russell, M. Talat Odman

**Affiliations:** † School of Civil and Environmental Engineering, Georgia Institute of Technology, Atlanta, Georgia 30332, United States; ‡ National Center for Environmental Health, Centers for Disease Control and Prevention, Atlanta, Georgia 30341, United States; § United States Department of Agriculture Forest Service, Pacific Northwest Research Station, Seattle, Washington 98103, United States

**Keywords:** wildfires, prescribed fires, postprescribed burn wildfires, smoke, CMAQ, BlueSky

## Abstract

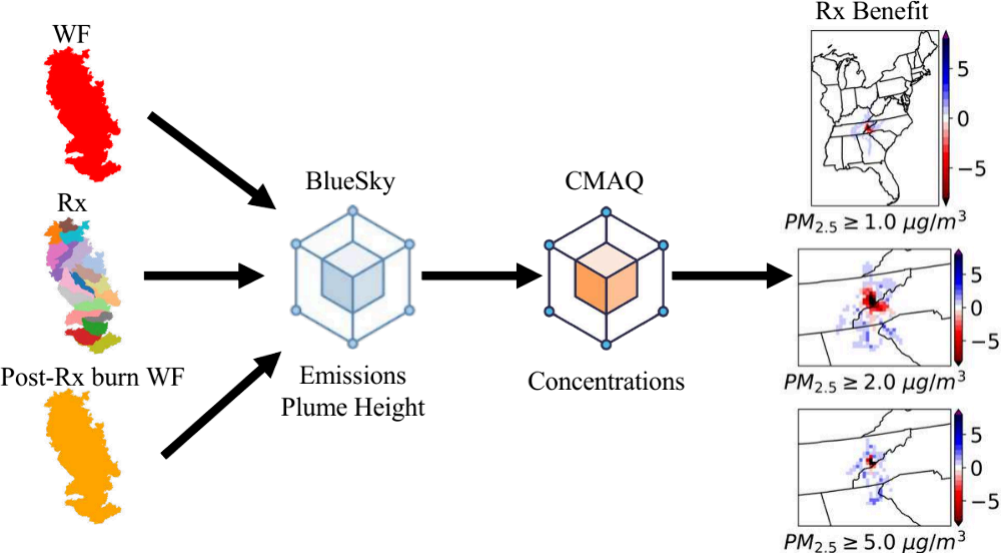

Prescribed burning is an effective land management tool that provides a range of benefits, including ecosystem restoration and wildfire risk reduction. However, prescribed fires, just like wildfires, introduce smoke that degrades air quality. Furthermore, while prescribed fires help manage wildfire risk, they do not eliminate the possibility of wildfires. It is therefore important to also evaluate fire and smoke impacts from wildfires that may occur after a prescribed burn. In this study, we developed a framework for understanding the air quality and health related trade-offs between wildfires and prescribed fires by simulating a set of counterfactual scenarios including wildfires, prescribed fires, and postprescribed burn wildfires. We applied this framework to the case of the Gatlinburg wildfire and found that emissions from prescribed burns and subsequent wildfire were slightly lower than those from the wildfire itself. This reduction resulted in lower daily average concentrations and exposures of PM_2.5_, O_3_, and NO_2_. Even considering the possibility of a postprescribed burn wildfire, prescribed fires reduced population-weighted daily average PM_2.5_, daily maximum 8-h average O_3_, and 1-h maximum NO_2_ concentrations. In Sevier County, Tennessee where the wildfire occurred, these reductions reached 5.28 μg/m^3^, 0.18 ppb, and 1.68 ppb, respectively. The prescribed fires also reduced the person-days smoke exposures from the wildfire. Our results suggest that although prescribed fires cannot eliminate the air quality impacts of wildfires, they can greatly reduce smoke exposure in downwind areas distant from the burn sites.

## Abstract 2

The work proposes a modeling framework which considers emissions from prescribed fires, wildfires, and postprescribed burn wildfires for understanding the air quality trade-offs between wildfires and prescribed fires.

## 1 Introduction

Prescribed fires are planned, controlled fires that have multiple benefits for the ecosystem health,^1^ hazard reduction,^2^ and endangered wildlife protection.^3^ Also, prescribed burning is a land management tool that can reduce the likelihood of catastrophic wildfires.^4,5^ However, both prescribed fires and wildfires emit significant amounts of pollutants such as particulate matter with aerodynamic diameter less than 2.5 μm (PM_2.5_), volatile organic compounds (VOCs), and nitrogen oxides (NO_*x*_) into the troposphere, and these pollutants have adverse health impacts.^6,7^ Recognizing that wildland fire management policies over the past century are not sufficient^8^ for wildfire risk reduction, and a warming climate is changing wildfire occurrence,^9^ it is increasingly important to evaluate prescribed fire and wildfire synergies and feedbacks in order to advise the prescribed fire management policy and evaluate the air quality-related benefits or burdens from prescribed burning decisions. Jaffe et al.^10^ compared the spatial and temporal patterns and emissions intensities of prescribed fires and wildfires in the U.S. The discrepancies in timing and locations for prescribed fires and wildfires make it challenging to compare the air quality impacts of prescribed fires with those of wildfires. Williamson et al.^11^ conceptually accounted for these differences by proposing a research framework around “smoke regimes” similar to how fire ecologists use fire regimes. While measurement^12−14^ and simulation^15−18^ methods are being applied to this challenging problem, the research community is still in search of suitable methods for reliable prescribed fire/wildfire air quality impact comparisons, such as the one provided in this study.

To effectively compare the impacts through measurements, it is essential to have a geographic area where both wildfires and prescribed burns frequently occur. In the southeastern U.S., high-intensity wildfires are rare, but in the western U.S., there are opportunities for conducting comparisons of wildfire and prescribed fire impacts. For example, Schweizer et al. analyzed the impacts of 14 fires, 7 high-intensity, and 5 low-intensity wildfires and 2 prescribed fires, in the California Sierra Nevada.^19^ Looking at increases in ground-level PM_2.5_ concentrations in population centers at distances of approximately 50 to 100 km on days with satellite-observed smoke aloft, they found that prescribed fires and low-intensity wildfires result in a much lower exposure per unit area burned than high-intensity wildfires. This study assumed that the fuel and atmospheric conditions are the same for different fires and the transport of smoke to populated areas is similar. Even if the first two assumptions hold, the distance from the fire matters as nearby fires would have a larger impact than more distant fires of the same intensity. Similar opportunities for comparing the impacts exist in other locations, for example, in Sydney, Australia, where wildfires and prescribed fires coexist.^20^

Navarro et al. took a different approach by reviewing reports of exposures to wildfire and prescribed fire smoke in the literature,^12^ and identified nine studies reporting smoke concentrations related to wildfires and seven related to prescribed fires. The review pointed to several issues with how smoke measurement methods are used in the analysis of impacts. In general, instruments measuring prescribed fire smoke are placed much closer to the fire than those reporting wildfire smoke. Consequently, the smoke measurements found in the literature for prescribed fires are relatively higher compared to their wildfire counterparts. Without regard to the proximity of the fires, this situation may lead to overestimation of the prescribed fire smoke impacts. While it is important to understand the local impacts of the prescribed fire smoke, it is also crucial to have accurate measurement further downwind to make informed decisions and distinguish air quality impacts between communities near the burn site and those in downwind areas. Huang et al., using low-cost sensors, have shown that existing PM_2.5_ monitoring networks are not capable of measuring the prescribed fire impacts in the southeastern U.S.^21^ Similarly, Marlier et al. pointed to the inadequacy of existing regional monitoring systems in identifying the exposure of outdoor agricultural workers to wildfire smoke in California.^22^ Gaps in ground observations can be partially bridged with satellite data, though this method is not without its limitations. First, not all fire plumes are visible to satellites, especially those from small, low-intensity prescribed fires.^23^ Then, satellites detect vertical column densities, and ground-level concentrations do not always correlate with the presence of smoke aloft; the plumes of some high-intensity wildfires may shoot up into the free troposphere and have little impact in the boundary layer over downwind population centers in close proximity.^24^ What is helpful is a combination of satellite data with models—whether statistical,^20^ machine learning,^25^ trajectory,^26^ or chemistry transport models (ref (27))—that can accurately relate to ground-level concentrations.

To understand the trade-offs of prescribed fires, fire emission models and air quality models are efficient tools since the trade-off scenarios typically include factual or counterfactual (or hypothetical) prescribed burns, postprescribed burn wildfires, or wildfires in unmanaged lands. One method is to compare the wildfire and counterfactual prescribed burns and assume the postprescribed burn wildfire does not happen. Kelp et al. conducted an adjoint chemistry transport model to simulate the prescribed fire and wildfire emission influence under different meteorological conditions.^16^ The prescribed burns were treated as replacements for wildfires. However, the timing of the prescribed burns was not well-designed, and the fire emission estimations were highly simplified (50% reduced emissions for prescribed burns in all landscapes). Kiely et al.^28^ employed a fuel load and fuel consumption estimation framework to improve the estimations of wildfire and prescribed fire emissions. The study indicated that the prescribed fires reduced the mortality due to PM_2.5_ exposure compared to wildfires. Wildfire ignitions may inevitably occur on landscapes treated with prescribed fire. However, prescribed fires may limit the area burned and reduce the fire severity of future wildfires, resulting in less fuel consumption, and hence emissions, than what would have occurred in the absence of the prescribed fire treatment. This potential reduction in future wildfire emissions, sometimes referred to as avoided wildfire emissions,^4^ must be estimated to quantify possible long-term net benefits to air quality. Jones et al. suggested evaluating the potential benefits of prescribed fires by considering the health impacts from high-intensity wildfires, prescribed burns, and low-intensity postprescribed burn wildfires.^29^ A recent example of simulation based comparative assessment by U.S. Environmental Protection Agency (EPA) focused on two wildfires in the western U.S., the Timber Crater 6 Fire that burned 3000 acres in Crater Lake National Park in 2018 and the Rough Fire that burned 150,000 acres in Sierra Nevada National Forest in 2015. Both fires’ scars included lands previously treated by prescribed fire. Hypothetical prescribed burn scenarios with more or less treatment and following postprescribed burn wildfire scenarios were simulated using a fire emissions estimation framework and a chemistry transport model. The results suggest that no prescribed burning would lead to the largest amount of PM_2.5_ exposures and increasing prescribed burning would reduce the PM_2.5_ exposures.^15^ Schollaert et al. designed prescribed fire management scenarios with different management extents.^30^ The study simulated several decades of prescribed fire decisions and considered the evolution of fuels by applying a landscape change model.^31^ The fire emissions estimated by a fuel type and combustion model were used in an inert tracer dispersion model. The study indicated that a scenario with moderate intensity of prescribed fire management had optimized health benefits. Afrin et al.^7^ evaluated the benefits of counterfactual prescribed burns for the prevention of two wildfires in North Carolina and considered postprescribed burn wildfire occurrence. The study revealed that the prescribed fires mitigated the PM_2.5_ exposures in the region. The emission calculations in this study used the size and centroid of the fires instead of the actual boundaries of fires, which is an oversimplification of the fuel load and emission estimations. Additionally, the counterfactual prescribed burns were conducted with equal size for each burn date, without accounting for the presence of firebreaks. The design of the prescribed fire perimeter can affect the magnitude and temporal patterns in emissions.

Ideally, the method used for assessment of air quality trade-offs must be free from any biases that may favor or discourage prescribed burning. However, in practice it is not easy to come up with such a method. Here, we focused on the Gatlinburg wildfire, which began in late November 2016. This wildfire was ignited by human error within the Great Smoky Mountain National Park near Gatlinburg, Tennessee prior to a big windstorm. It grew dramatically, burning over 10,000 acres, and impacted Gatlinburg and nearby communities. The fire resulted in 14 fatalities, including two deaths directly or indirectly due to the inhalation of smoke,^32^ loss of more than 2000 structures, and more than 2 billion dollars of damage.^33^ Our study implemented a new framework for designing counterfactual prescribed fires with considerations of fire behavior and meteorological conditions. Also, instead of treating each fire as a point for estimation of emissions, as done in some previous studies,^7,28^ we implemented an algorithm for designing the perimeters of counterfactual prescribed burns, which can also be applied in future trade-offs studies. Then, we used the boundaries of the fire to improve the fuel representation accuracy. The estimates for wildfire, prescribed burns, and postprescribed burn wildfire emissions were applied to simulate the air quality impacts with a chemical transport model. The population-weighted concentrations and the population impacted by fire smoke under different fire scenarios were analyzed to evaluate the trade-offs between wildfires and prescribed fires. The framework implemented in this study can be applied to other wildfire-prescribed fire trade-off case studies.

## 2 Methods

In this study, we focused on the main part of the Gatlinburg wildfire (WF) located in Sevier County, Tennessee, U.S (Figure S1). The fire burned from Great Smoky Mountain National Park and headed to the Gatlinburg area and Pigeon Forge City. We designed three scenarios to evaluate the emissions and air quality trade-offs between prescribed fires and wildfires (Table 1). The counterfactual prescribed burns (Rx) were designed with consideration of firebreaks and meteorological conditions. We also assumed a postprescribed burn wildfire (post-Rx WF) occurrence with reduced fuel load after prescribed fire consumptions. We estimated emissions for different scenarios with fires at the Gatlinburg wildfire region. The smoke impacts were estimated by subtracting no-fire baseline simulations from the simulations with fires. The differences between smoke impacts from Scenario 2 and Scenario 3 were used to assess the benefits or losses of the prescribed fires considering the postprescribed burn wildfire occurrence.

**Table 1:** Simulation Scenarios

**Scenarios**	**Scenario descriptions**
Scenario 1 (baseline)	No counterfactual prescribed burns (no Rx)
	No Gatlinburg wildfire (no WF)
Scenario 2 (factual wildfire)	No Rx
	WF (wildfire case)
Scenario 3	Rx: 19 prescribed burns under favorable meteorological conditions before WF (prescribed fire case)
	Postprescribed burn wildfire (Post-Rx WF) with reduced fuel loads during WF period (postprescribed burn wildfire case)

### 2.1 Design of Counterfactual Prescribed Fires

#### 2.1.1 Selection of Prescribed Fire Dates

Prescribed fires are conducted under meteorological conditions favorable for safe ignition and controllable fire propagation that also minimize local smoke impacts. Fire managers typically use weather forecasts to decide whether to conduct prescribed fires or not. In this study, we used the following rules from prescribed burning management guidelines for date selection: 24-h rain <6.35 mm/day (0.25 in./day), relative humidity >30%, temperature <29.4 °C (85 °F), planetary boundary layer between 503 and 1981 m (1650 to 6500 feet), wind speed between 3.6 and 6.3 m/s (8 to 14 mph), and transport wind speed between 4.0 and 8.9 m/s (9 to 20 mph).^34^ We obtained the meteorological conditions from our Weather Research and Forecasting Model (WRF, version 3.9)^35^ simulation for a grid cell near Gatlinburg (Figure S1).

#### 2.1.2 Determination of Prescribed Fire Boundaries

Firebreaks are natural or manmade obstacles that keep the prescribed fires from escaping out of the designated burn units. The boundaries of the burn units are designed to allow the prescribed fires to be conducted safely and reduce the cost of building firebreaks.^36^ In this study, we first used the 30-m resolution National Land Cover Database (NLCD) 2016^37^ to find existing firebreaks such as barren land, open water, or developed urban regions (roads or railways)^36,38^ (Figure S2). The risk management practices suggest limiting the burned area to less than 1000 acres per day.^39^ However, some prescribed fire units with boundaries designed from existing firebreaks had areas larger than 1000 acres. We further split those large units by considering fire behavior over sloped terrain. Fire propagates faster when the slope is steeper due to the preheating effects.^40^ We utilized terrain slope data from 30-m resolution Landscape Fire and Resource Management Planning Tools (LANDFIRE)^41^ products and set boundaries where the slope is gentler (<20 degrees) to allow more control over the spreading rate of prescribed fires (Figure S3). For extracting the boundary line that follows the target type of grid cells (grid cells with desired land cover type for NLCD data and with gentle slope values for LANDFIRE data) in the raster data sets, which are composed of grid-based data that includes geographic attributes, we implemented an algorithm combining the dilation operation^42^ and uniform-cost search.^43^ The dilation operation simplified the boundary of grid cells with target values, and the uniform-cost search found a path that follows the target grid cells between a start point and an end point (algorithm S1).

### 2.2 Wildfire and Prescribed Fire Emissions

We used the BlueSky framework (version 4.3)^44^ to estimate emissions of the Gatlinburg wildfire, counterfactual prescribed burns, and postprescribed burn Gatlinburg wildfire. BlueSky incorporates modules to estimate fuel type, fuel load (FCCS module^45^), fuel moisture (NFDRS module^46^), and fuel consumption (CONSUME module^47^), sequentially (Table S1). Then, it combines this information with Prichard-O’Neill emission factors to derive the fire emissions.^48^ The smoke plume will reach different heights and have different vertical structures depending on the type of fire due to the differences in heat fluxes emitted from wildfires and prescribed fires.^49,50^ We utilized the Briggs plume height model, which uses the estimated heat from the consumption model to estimate the plume vertical structures.^51^ Fuels are spatially heterogeneous. The BlueSky utilized a 1-km resolution FCCS fuel map to estimate the fuel type and fuel load. For improved estimations of fuel type and fuel load for fires in our study, we used the fire boundaries in the BlueSky framework instead of assigning the values at the centroids of burned areas. To consider the differences in fuel load, consumption, and emissions among prescribed burns, the wildfire, and the postprescribed burn wildfire, we conducted different configurations in different scenarios. We ran BlueSky with wildfire mode for the wildfire and the postprescribed burn wildfire, which consumed canopy fuels and applied wildfire emission factors. The postprescribed burn wildfire had the same fire boundary and burned date as the Gatlinburg wildfire, while the postprescribed burn wildfire fuel load was reduced based on fuel consumption from counterfactual prescribed burns. However, we assumed no regrowth of understory vegetation after the prescribed fire treatment for the postprescribed burn wildfire case. For the prescribed fire case, the emissions were estimated in BlueSky’s prescribed fire mode without canopy consumption since prescribed fires typically consume the understory. The detailed BlueSky settings can be found in Supporting Information (Table S1).

### 2.3 Air Quality Simulations

**Figure 1:**
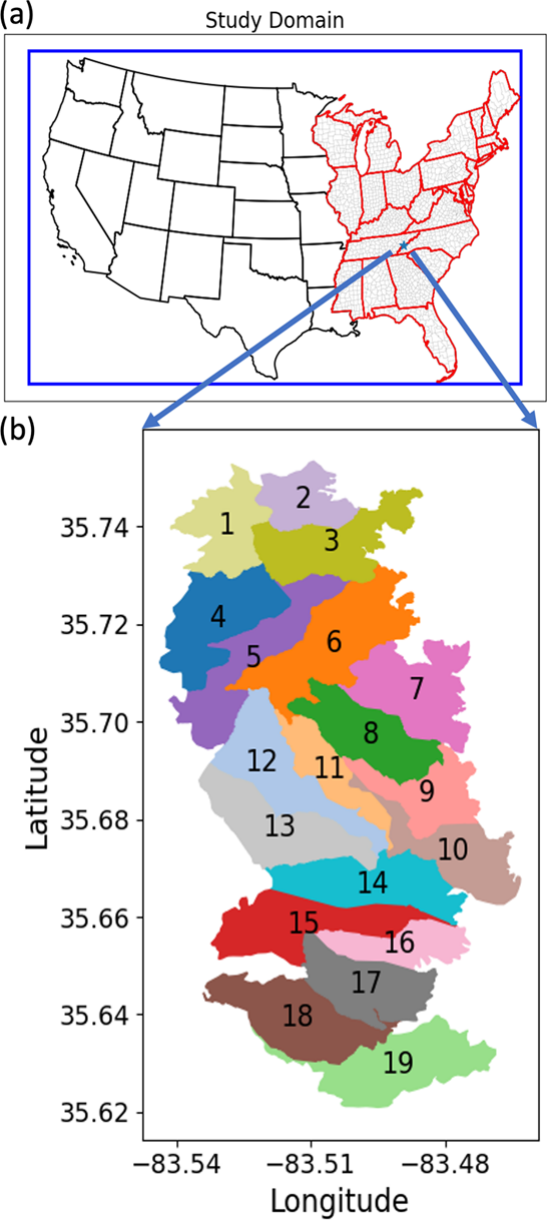
(a) CMAQ 12-km resolution domain. The blue box is the CMAQ simulation domain with 246 rows and 396 columns. The red boundaries show the states focused in the study. The blue star shows the Gatlinburg wildfire location. (b) Boundaries of counterfactual prescribed burns. Each prescribed burn’s boundary is filled with a different color. The number in each boundary indicates the burn number assigned for each prescribed burn. The burn date and the burned area associated with each burn number are shown in Table S4.

We used the Community Multiscale Air Quality (CMAQ, version 5.4) modeling system,^52^ an Eulerian chemical transport model (CTM), to simulate the air pollution concentration under different scenarios. The meteorological conditions for the model were provided by the WRF, version 3.9.^35^ The CMAQ model was under 12-km resolution, and the domain covered the contiguous United States (CONUS) (Figure 1). We used the Carbon Bond 6 (CB6) gas phase chemistry mechanism and the AERO6 aerosol module in CMAQ to represent the evolution of primary pollutants and the formation of secondary pollutants. The National Emission Inventory (NEI)^53^ was applied for all anthropogenic and wildland fire emissions, excluding the Gatlinburg wildfire. The biogenic emissions were calculated online by the Biogenic Emission Inventory System, version 4 (BEIS4)^54^ included in CMAQ. For the baseline scenario, we included all anthropogenic, wildland fire, and biogenic emissions except the fire emissions related to the Gatlinburg wildfire. For the other two scenarios, the corresponding fire emissions from BlueSky were input as grid-based emissions with 3D structures considering the Briggs model estimated plume height and plume bottom with uniform vertical profile assumption along with all other emissions.

We conducted continuous CMAQ simulations, beginning 2 days prior to each fire case for model spin-up and extending 5 days after the final burn date to capture postburn smoke transport. Since smoke transport within the study domain typically lasts less than 2 days, our analysis focused on concentrations and exposures for the burn dates and the subsequent 2 days. Table S6 provides the specific CMAQ simulation and analysis periods used in this study. The daily average PM_2.5_, daily maximum 8-h (MDA8) O_3_, and 1-h daily maximum NO_2_, which have standards in National Ambient Air Quality Standards (NAAQS), were the pollutants discussed in this study. The performance of CMAQ model in simulating these pollutants under the factual scenario (Scenario 2) was evaluated by comparing the modeled concentrations with EPA monitoring data^55^ using statistical metrics whose formulas are given in Text S1, such as normalized mean bias (NMB), normalized mean error (NME), and Pearson correlation coefficient (also known as R).

### 2.4 Population-Level Exposure Ascertainment

We simulated three scenarios using CMAQ v5.4^52^ with different fire emissions. The impacts of fires in Scenarios 2 and 3 were estimated by subtracting the concentration of the baseline (i.e., no-fire) scenario (Scenario 1). To understand the health impacts of prescribed fires, postprescribed burn wildfires, and wildfires, we first created a population-weighted measure of pollutant concentration, which is defined as
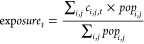
where *c*_*i,j,t*_ and *pop*_*i,j*_ are respectively the fire-associated daily pollutant concentration on day *t* and population for each ground-level grid cell (*i,j*) in CMAQ simulations. For population data, we used 1-km resolution population data for the United States^56^ and regridded it onto CMAQ grids by using the nearest neighbor approach (Figure S11).

In our study, we used the concept of person-days (PD) to quantify the population exposed to smoke under different fire events.^57^ Person-days refers to the cumulative time that individuals in a population are exposed to a certain pollutant concentration level, offering a useful metric for evaluating trade-offs between prescribed fires and wildfires. This metric accounts for both pollutant concentrations and exposure duration, effectively capturing the differences in exposure time between prescribed fires and wildfires. Person-days can be mathematically defined as
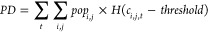

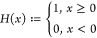
where *c*_*i,j,t*_ and *pop*_*i,j*_ maintain the same definition as before. *H*(*x*) is a unit step function^58^ and it is used to count the population in grid cell (*i,j*) when the associated concentration exceeds the selected threshold for each day. We first accumulated the counted population among all the ground-level grid cells over the study domain to evaluate the daily person-days under different pollutant levels. Then, we summed up the daily person-days over the scenario’s time period to calculate each scenario’s person-days. The benefit from prescribed fire is the person-days difference between wildfire (Scenario 2) and the sum of prescribed burns and the postprescribed burn wildfire (Scenario 3).
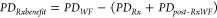
where *PD*_*WF*_, *PD*_*Rx*_, *PD*_*post-Rx WF*_ are the total person-days from wildfire, prescribed burns, and postprescribed burn wildfire cases. *PD*_*Rx benefit*_ denotes the benefits from prescribed burns and the positive *PD*_*Rx benefit*_ mean the prescribed fires prevented exposures.

## 3 Results

### 3.1 Designed Prescribed Fires

Before the wildfire occurrence, we selected 19 days under favorable prescribed fire weather conditions to conduct the designed prescribed burns (Figure 1). For each prescribed burn, the boundaries of the burn area were created based on the existing firebreaks, such as developed regions and barren land from NLCD or gentle sloped regions from the LANDFIRE product, to ease control of the fire. The algorithm we designed (Algorithm S1) guarantees the boundaries of the prescribed fires align with the desired firebreaks or topographical features (Figures S4 and S5). All the burn areas were limited to be less than 1000 acres except for one prescribed fire on January 14th, 2016, which had a burn area of 1233 acres. Also, we designed a “zigzag” burning sequence to execute the prescribed fires in an orderly fashion, reducing the distance between the burns.

### 3.2 Wildfire, Prescribed Fires, and Postprescribed Burn Wildfire Emissions

**Figure 2:**
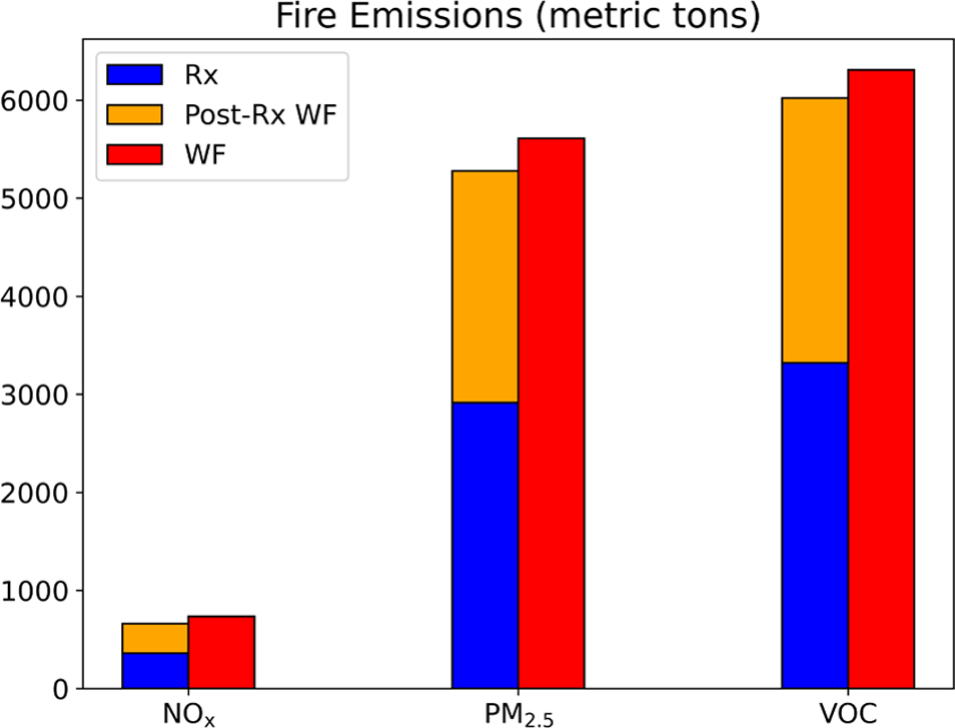
Total emissions of NO_*x*_, PM_2.5_, and VOC in metric tons under the wildfire, prescribed burn, and postprescribed burn wildfire cases for the Gatlinburg wildfire region.

Fuel Characteristic Classification System version 2 (FCCS) fuel map^45,59^ incorporated in the BlueSky fuel model provided the fuel type and fuel load in the Gatlinburg wildfire region (Figure S6). The fuel type data indicated that 51.7% of vegetation was yellow poplar/sugar maple/basswood forest, and the rest was chestnut/white northern red oak forest. The fuel consumption estimates from the CONSUME model are shown in Table 2. The prescribed fires consumed a total of 179,779.2 tons of fuel, and the wildfire consumed 356,483.3 tons. The main differences in fuel consumption between prescribed fires and wildfires were in canopy consumption, where prescribed fires, as low-intensity and controlled fires, typically consumed the ground fuels such as duff. Since the postprescribed burn wildfire happened after the prescribed fire treatment, we subtracted the fuel consumed in prescribed burns from the fuel load in the Gatlinburg wildfire region with the prescribed fire fuel consumption before the postprescribed burn wildfire BlueSky simulations. Also, we reduced the canopy consumption from 50% to 20% in postprescribed burn wildfire, considering the intensity of the fire would be reduced due to lighter fuel loads. The total consumption of postprescribed burn wildfire was 147,476.2 tons, corresponding to a 58.6% decrease compared to the wildfire. The largest decrease in fuel consumption was from canopy by 99,261.0 tons. Then, the emissions were estimated based on the fuel consumption and corresponding emission factors for wildfires or prescribed fires. The total emissions of different fire cases are shown in Table S2, Figure 2, and the emissions for each designed prescribed burn are shown in Figure S7. The sum of NO_*x*_, PM_2.5_, and VOC emissions from prescribed burns and the postprescribed burn wildfire are 10.2%, 5.9%, and 4.6% less than the wildfire emissions, respectively. All fires emitted significant amounts of particulate matter (PM) and VOCs but limited amounts of NO_*x*_. Then, the fire emissions are distributed with empirical time profiles incorporated in BlueSky to provide hourly emissions for CTM (Figure S8, S9). For prescribed burns, we assumed the ignition starts at 10:00 (after the sunrise) and ends before 17:00 (before the sunset) local time (Figure S8). For wildfire, we simulate the fire emissions from Nov 25th, 2016 to Nov 29th based on the start and end times of NEI point emission records. BlueSky assumed the same diurnal profile (Figure S9) and equally distributed the burned area to each day. We used the Briggs plume height model to estimate the hourly vertical plume structures based on the input meteorological conditions and heat flux provided by the CONSUME model (Figure S10). The maximum plume height of wildfire is 6338.3 m, which is higher than the maximum postprescribed burn wildfire plume height (4188.9 m) and much higher than the maximum prescribed fire plume height (3294.7 m). This can be explained by the higher heat flux released from the wildfire. Meanwhile, the average prescribed fire plume height (2099.0 m) is higher than the average wildfire (1854.1 m) and postprescribed burn wildfire plume height (1250.6 m). This is due to the differences in temporal patterns of prescribed fires and wildfires. Prescribed fires typically start after sunrise and are completed before sunset, while the wildfires can last during the night and early morning. The lower planetary boundary layer (PBL) and moist fuel conditions at night reduce the fire plume heights and lead to lower average plume heights in the wildfire case.

**Table 2:** Fuel Load and Consumptions (Unit: Metric Tons) under the Wildfire, Prescribed Burn, and Postprescribed Burn Wildfire Cases for the Gatlinburg Wildfire Region

	**Canopy**	**Ground fuel**	**Litter-lichen-moss**	**Nonwoody**	**Shrub**	**Woody fuels**	**Total**
Fuel load	425704.8	94784.2	75088.2	3229.0	74999.0	120832.9	794638.1
WF consumption	165219.9	14607.9	51946.0	3136.4	48547.4	73025.7	356483.3
Rx burn consumption	0.0	8116.2	51776.4	3137.3	47682.9	69066.4	179779.2
Post-Rx WF consumption	65959.0	13985.6	15859.5	89.6	18488.2	33094.3	147476.2

### 3.3 Modeled Pollutant Concentrations

**Figure 3:**
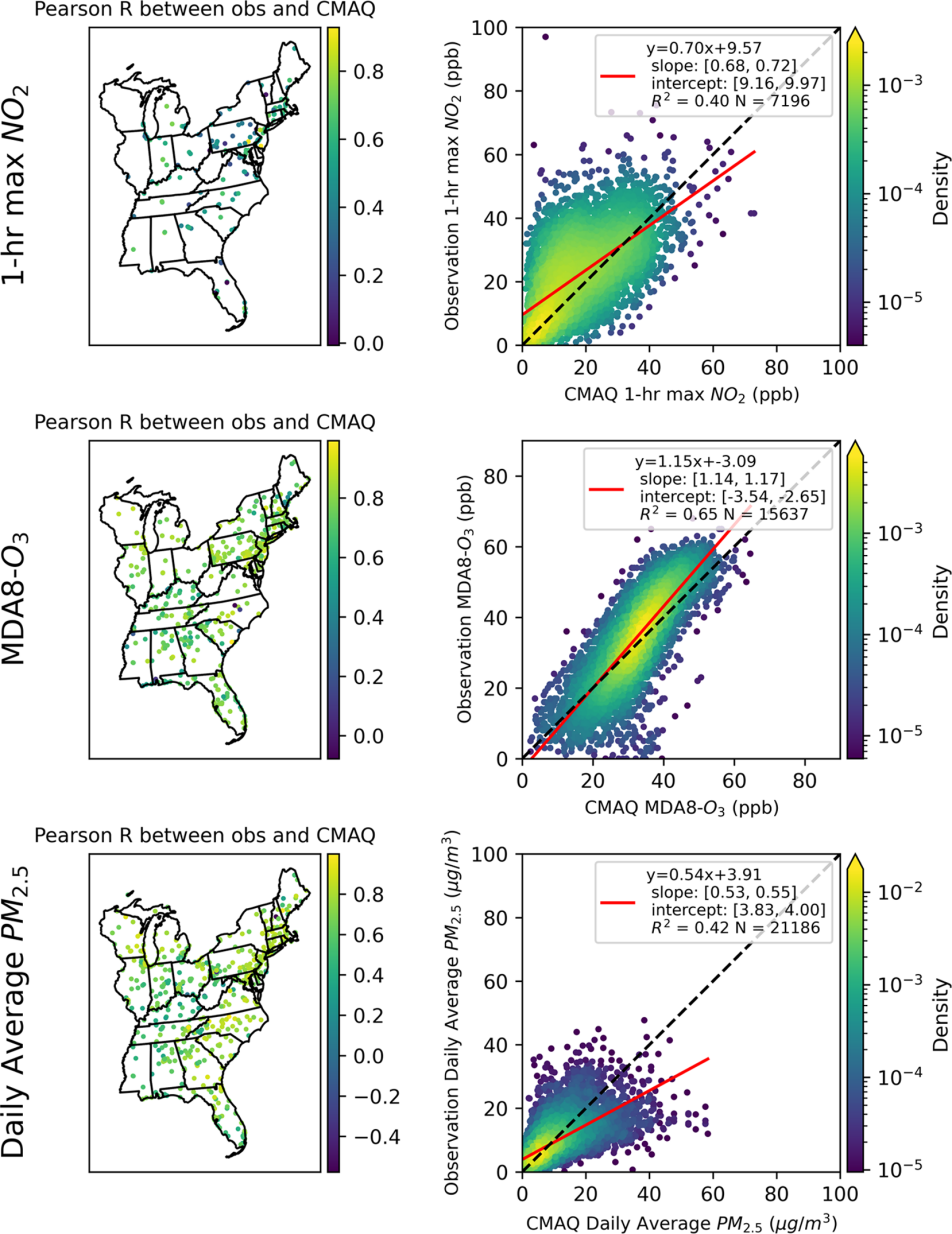
Model evaluation by comparing simulations to observations for daily average PM_2.5_, MDA8-O_3_, and 1-h max NO_2_ (the wildfire and counterfactual prescribed fire burn dates and the following 2 days). The spatial plots on the left show the Pearson correlation coefficient (R) value between simulation and observation for each monitor in the study domain. The density scatterplots on the right show the relationship between all observations and simulations. The black dashed line is the unity (1:1) slope line. The red line shows the linear relationship between simulation and observations. The R^2^ performance and 95% confidential interval of slopes and intercepts of the regression line are indicated. N shows the total number of data points in the linear regression.

Figure 3 shows the CMAQ model performance in the evaluation periods which include the no-fire scenario covering the date of designed prescribed burns and the subsequent 2 days (totaling 45 days), and the Gatlinburg wildfire scenario which burn period spans from November 25, 2016 to November 29, 2016 plus the following 2 days (totaling 7 days). For the daily average PM_2.5_, the NMB was −1.85%, the NME was 36.78%, and the R was 0.65. The 1-h daily maximum NO_2_ evaluation showed a −21.24% NMB, 38.70% NME, and an R value of 0.63. For the daily maximum 8-h ozone, the NMB was −5.69%, NME was 14.33%, and R was 0.81. The formulas, performance for all statistical metrics used in model evaluation, and detailed performance evaluation results for the 45-day period which covers the designed counterfactual prescribed burns and the following 2 days and the 7-day period which covered the Gatlinburg wildfire are available in Supporting Information (Text S1, Table S3, Table S6, S7, Figure S19, S20). The model performance was better than the recommend benchmark criteria suggested by Emery et al.^60^ Overall, the model underestimated NO_2_ and PM_2.5_ while it overestimated the ozone based on slopes of the regression lines (Figure 3). This is due to comparing well-mixed averaged grid cell values with point measurements. The concentrations of primary PM_2.5_ and NO_2_ can be greatly diluted. At the same time, the diluted NO_*x*_, which leads to less titration, can enhance the simulated ozone formation.^61^

**Figure 4:**
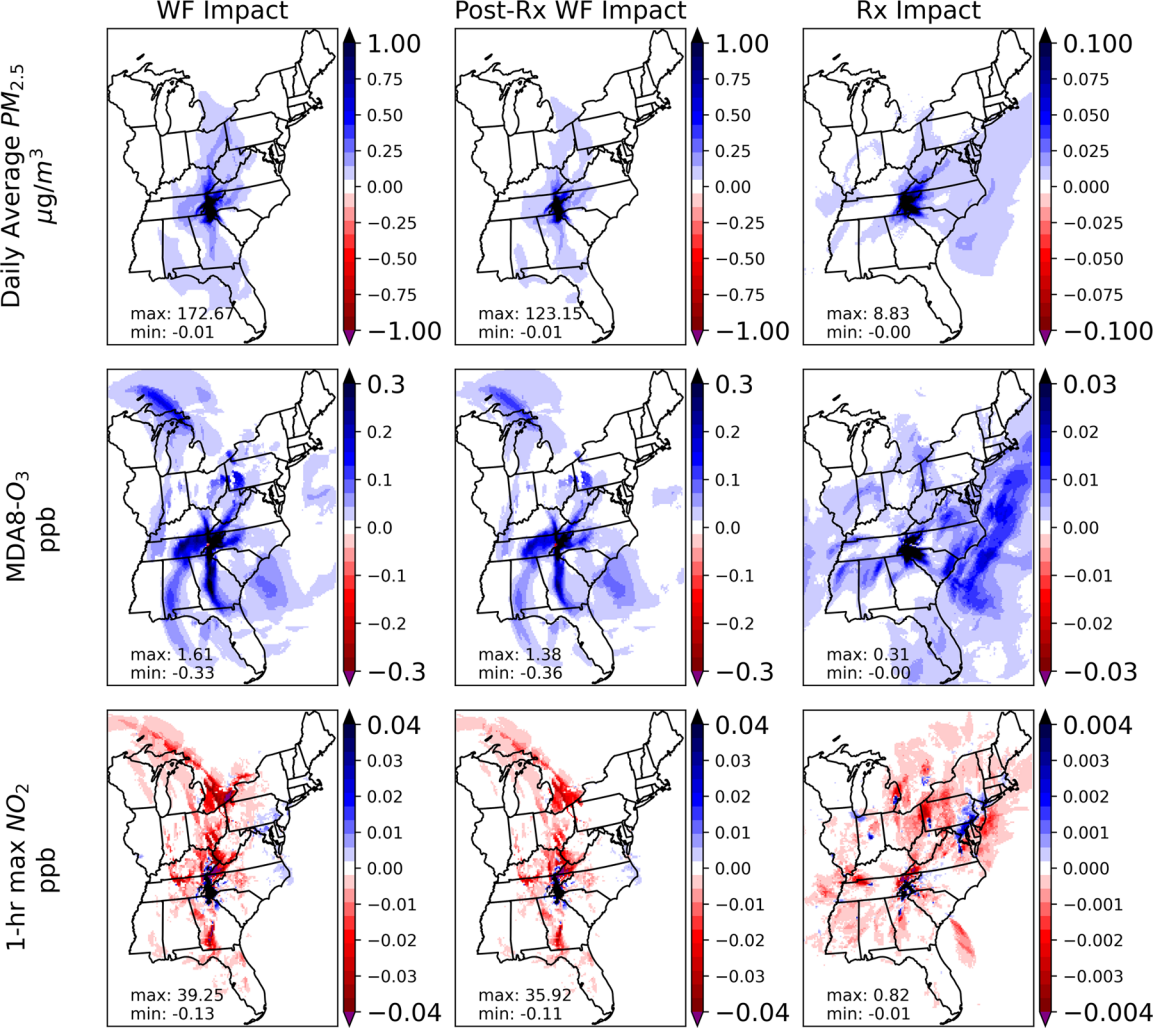
Mean daily average PM_2.5_, MDA8-O_3_, and 1-h max NO_2_ of smoke impacts during the wildfire, postprescribed burn wildfire, and prescribed burns periods. The smoke impacts are calculated by subtracting the baseline scenario concentrations from fire cases. The impact scale of prescribed burns is an order of magnitude lower than that of the other cases. Figure S21 shows the impacts on the same symmetrical log scale for all three types of fires.

Simulations show that the wildland fire emissions highly impacted the air quality during the fire periods. The spatial distributions of the mean concentration of PM_2.5_, MDA8-O_3_, and 1-h max NO_2_ during the fire dates are shown in Figure 4. The figure illustrates how different the smoke dispersion patterns were between the wildfire and prescribed fire cases. The Rx Impact has greater dispersion over the Atlantic Ocean, while the WF Impact had more north/south dispersion. It should be noted also that the scale of the Rx Impact panels is an order of magnitude less than the WF Impact panels. Finally, differences in dispersion patterns between the PM_2.5_ and O_3_ panels illustrate the complexity of atmospheric chemistry processes. Secondary processes are forming ozone and forming/reducing PM_2.5_ as the plume travels and interacts with anthropogenic and biogenic species. For the Gatlinburg wildfire, Tennessee was the state with the highest impact. The state-averaged increases in PM_2.5_, MDA8-O_3_, and 1-h max NO_2_ were 0.64 μg/m^3^, 0.14 ppb, and 0.11 ppb, respectively. Sevier County in Tennessee, where the wildfire occurred had the highest impacts from PM_2.5_ and NO_2_ due to the wildfire emissions where PM_2.5_, 1-h max NO_2_ increases were 26.10 μg/m^3^ and 6.49 ppb, respectively. However, Swain County in North Carolina had the highest increase in MDA8-O_3_ by 0.98 ppb since ozone formation is not necessarily local to the fire, depends on the presence of precursors in the area, and takes time. The NO_*x*_-limited smoke formed ozone when the smoke was transported to regions with high NO_*x*_ concentrations, such as urban areas. The impacts from postprescribed burn wildfire had a similar spatial distribution as the Gatlinburg wildfire case since these fire simulations were conducted during the same period with the same meteorological conditions, but with much lower smoke impacts. Again, Tennessee had the highest impact, with 0.46 μg/m^3^, 0.11 ppb, and 0.08 ppb increases in PM_2.5_, MDA8-O_3_, and 1-h max NO_2_, respectively. Sevier County’s PM_2.5_ and 1-h max NO_2_ increased by 18.56 μg/m^3^ and 5.41 ppb, respectively. Swain County had a 0.84 ppb increase in MDA8-O_3_. The counterfactual prescribed burns had the lowest impacts among all cases due to their lower emissions. Tennessee was still the state with the highest impacts of daily average PM_2.5_ and 1-h max NO_2_, which increased by 0.06 μg/m^3^ and 0.002 ppb, respectively, while North Carolina had the highest MDA8-O_3_ increase by 0.02 ppb. For the county averaged concentration, Sevier County still suffered from high PM_2.5_ and NO_2_ increasing due to the distance to the source, with 2.34 μg/m^3^ and 0.14 ppb increases, respectively. Jackson County in North Carolina had the highest MDA8-O_3_ impact with a 0.24 ppb increase.

### 3.4 Population-Weighted Concentrations and Person-Days

We estimated the averaged population-weighted concentration and person-days by considering the burn dates and the following 2 days (7 days included for WF and Post-Rx WF cases and 45 days included for the Rx case) since this period covers the smoke trajectories in the study domain. The mean population-weighted concentrations of wildland fire emissions under different fire cases were low over the whole study domain (PM_2.5_ < 0.07 μg/m^3^; MDA8-O_3_ < 0.04 ppb; 1-h max NO_2_ < 0.01 ppb). However, localized smoke exposures still raised concerns regarding smoke impacts (Figure S12). Tennessee, where the wildfire was located, had the highest population-weighted concentrations. The wildfire increased the state mean population-weighted exposure to PM_2.5_ by 0.49 μg/m^3^, MDA8-O_3_ by 0.13 ppb, and 1-h max NO_2_ by 0.07 ppb. For the prescribed burns case, Tennessee had the highest PM_2.5_ and NO_2_ exposures. The averaged population-weighted concentrations during considered periods were 88.70% and 96.42% less compared to the wildfire case. South Carolina had the highest MDA8-O_3_ exposures due to the prescribed burns, which was as much as 0.02 ppb. Ozone mostly impacted population in Greenville, a city near the border of North Carolina and South Carolina. To understand the prescribed fire-prevented population-weighted concentration, we calculate the differences between the wildfire scenario (Scenario 2) and the sum of the prescribed burns and postprescribed burn wildfire cases (Scenario 3) (Figure S13). The average population-weighted concentrations were much lower under Scenario 3 than Scenario 2, which were 48.97%, 46.15%, and 42.86% less. The effect of the prescribed fires was to reduce net population-weighted concentration relative to the actual wildfire event, except in areas immediately downwind during the prescribed fire events (along a strip extending from Tennessee to South Carolina) but upwind during the wildfire (Figure S13).

**Figure 5:**
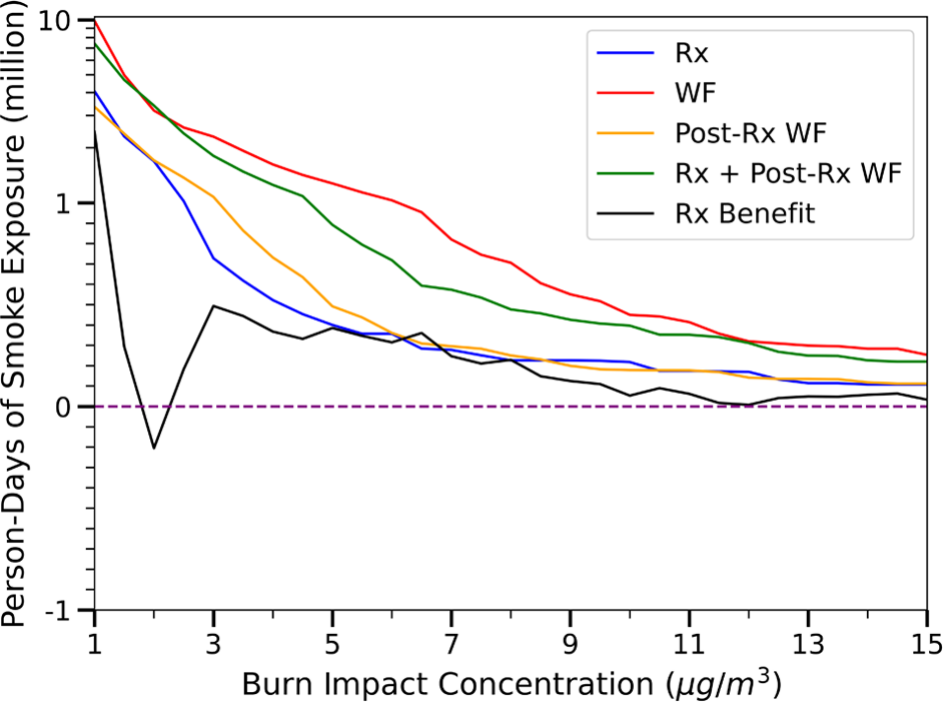
Person-days under Gatlinburg wildfire (WF) and prescribed fire (Rx), and postprescribed burn wildfire (Post-Rx WF) cases of Scenario 3 for specific burn impact concentration thresholds, represented by red, blue, and orange lines, respectively. The green line shows the sum of person-days for scenario 3. The black line shows the person-days prevented by prescribed fires Rx, calculated as the difference in person-days of exposure between the Gatlinburg wildfire and Scenario 3. The dashed line represents zero person-days. When the black line is above the dashed line, the combined exposure from prescribed fire and postprescribed burn wildfire is smaller than wildfire exposure.

Since PM_2.5_ was the most impacted pollutant in the previous analysis, we calculated the person-days for burn impact PM_2.5_ concentration between 1 μg/m^3^ and 15 μg/m^3^ to capture the different extent of exposures (Figure 5), as most of the population in the study area is impacted within this range. For the select ranges, the prescribed burn and postprescribed burn wildfire person-days were lower than wildfire. The prescribed fire has net benefits when the benefits line (red line in Figure 5) is above zero. In this study of the Gatlinburg wildfire, prescribed fire effectively prevented the PM_2.5_ exposures under person-days criteria for most of the burn impact concentration thresholds except at a PM_2.5_ impact level near 2 μg/m^3^ (Figure 5).

**Figure 6:**
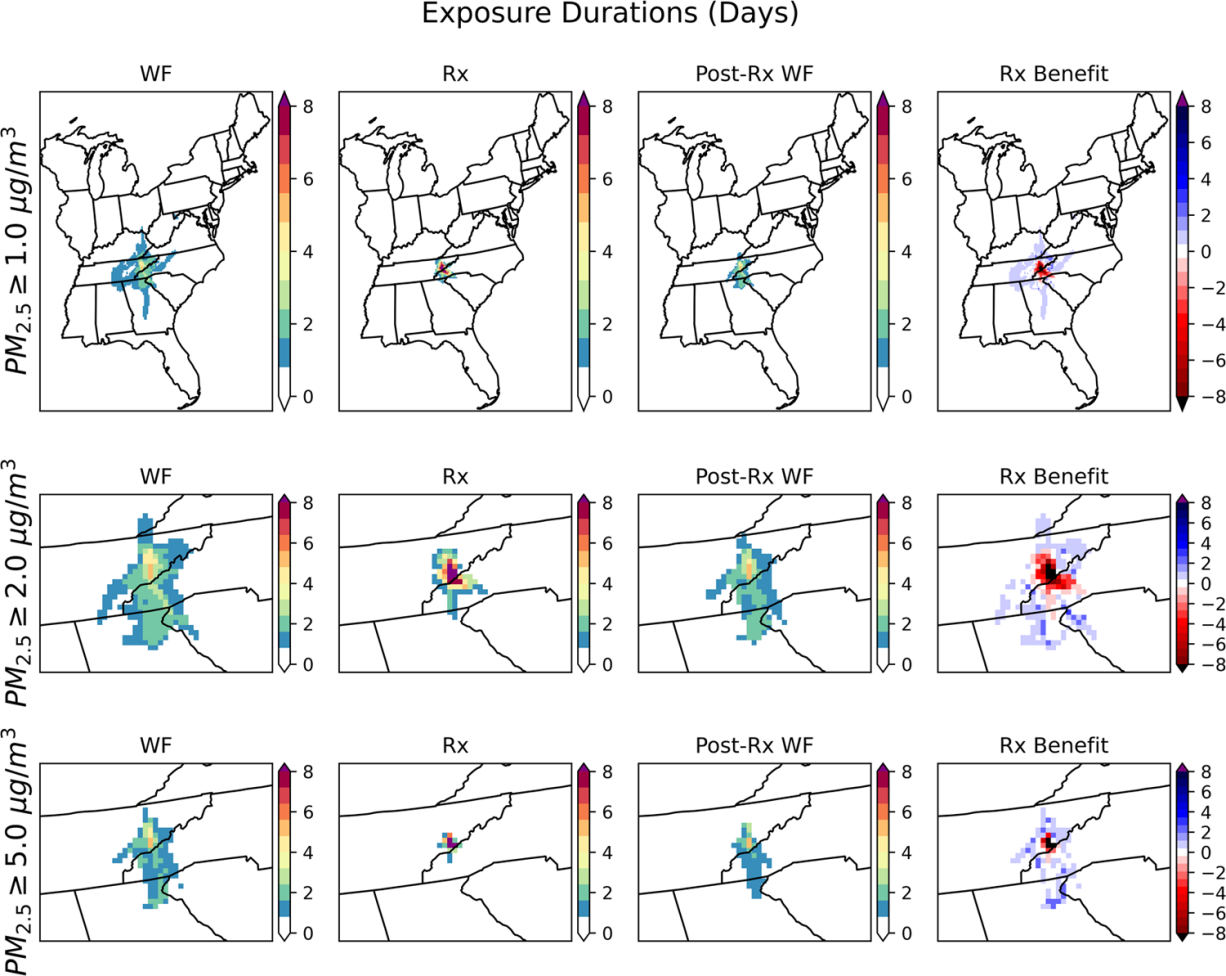
Exposure durations when the PM_2.5_ concentration is higher than 1.0 μg/m^3^ (top), 2.0 μg/m^3^ (middle), and 5.0 μg/m^3^ (bottom). For PM_2.5_ ≥ 1.0 μg/m^3^, the spatial domain shown is the entire study domain. For the PM_2.5_ ≥ 2.0 μg/m^3^ and PM_2.5_ ≥ 5.0 μg/m^3^, the domains shown cover the Tennessee-North Carolina border since PM_2.5_ impacts are local. The Rx benefit or Rx-prevented exposure duration is calculated by WF exposure duration minus Rx exposure duration minus post-Rx WF exposure duration.

For low levels of PM_2.5_ (PM_2.5_ ≤ 1 μg/m^3^) impact, prescribed fires have positive benefits of preventing population exposures. The wildfire smoke, which had higher energy and higher plume height, was transported by stronger winds during the daytime, and induced a larger range for smoke impact (Figure 6, top). For the prescribed fire with lower emissions and lower plume height in the daytime, the smoke impact was concentrated near the fire region. The long-range transported smoke in the wildfire case easily reached the selected low-level thresholds (1 μg/m^3^), which led the populations in Tennessee and its nearby states, including Alabama, Georgia, South Carolina, North Carolina, Virginia, and Kentucky, to be exposed. The prescribed burns with smaller spatial air quality impacts reduced the overall smoke exposure resulting from both wildfire and postprescribed burn wildfire. Prescribed burning led to a disbenefit at or above 2 μg/m^3^. This can be explained by the long-distance transport of wildfire smoke, which affected the nearby states, being mostly lower than 2 μg/m^3^, and exposure near Gatlinburg was the dominant concern. Prescribed burns that covered a longer period than the wildfire induced higher person-days around Gatlinburg (Figure 6, middle). Regional analysis of different levels of person-day exposures (Figure S14, S15) also indicated that Tennessee, where the wildland fires occurred, and neighboring North Carolina experienced negative impacts while Georgia benefited from prescribed burning. Prescribed fires prevented exposures to PM_2.5_ concentration ≥5 μg/m^3^ almost everywhere expect in Gatlinburg, because, with their lower emissions, their high concentration impacts cannot reach to long distances (Figure 6, bottom). Also, the nighttime smoke in the wildfire scenario increased the smoke concentrations near the fire region, while prescribed fires which ended before sunset prevented nighttime exposures.

## 4 Discussion

Prescribed fire is a widely used land management tool primarily aimed at reducing hazardous fuel loads and restoring and maintaining ecosystem health. An additional benefit of prescribed fire is its potential of reducing smoke impacts even if wildfires occur in previously prescribed burned areas. In this study, we implemented a new framework to evaluate the air quality trade-offs between prescribed fires and wildfires and found that prescribed fires have additional benefits to mitigate the smoke impacts from the Gatlinburg wildfire, even considering the occurrence of a postprescribed burn wildfire. The prescribed burn units were designed with consideration of firebreaks in our modeling framework. Fire emissions were estimated taking into account the boundaries of the fires. Using the fire boundaries to estimate the emissions instead of the centroids of the fires and fire sizes can better represent the fuel type and fuel load, which are critical factors for emissions estimation. Also, the firebreaks design method provided more realistic estimations of the burned area instead of making simple assumptions such as equally distributing the burned area among the prescribed burns. Although the total emissions can be similar for a different prescribed fire design, such as a grid-based boundary design (Figure S16), downwind smoke concentrations can vary due to different daily emission distributions (Figure S17). The emission distribution under grid-based boundary design led to a slightly increased population-weighted concentration due to higher smoke impacts in Georgia, where the population density is higher than South Carolina, which suffered more smoke under prescribed burns designed considering firebreaks.

For the design of counterfactual prescribed burns, the meteorological conditions are other important factors to be considered. Although it is possible to relax the criteria for burning and find potential burn days with less probability of smoke transport to populated areas,^28^ this may raise additional concerns. First, the meteorological conditions are critical for fire spread and fire control. For instance, if the winds are very strong and the fuels are excessively dry, there is a risk of prescribed fires escaping control and evolving into wildfires. Conversely, if the winds are weak and the fuels are wet, then ignition becomes challenging. Additionally, selecting the dates when the smoke is not transported to populated areas increases the likelihood that the smoke impacts will be felt in areas with lower population density. These are usually rural areas with low economic status and limited access to health care, entailing environmental justice and equity concerns. In this study, we assumed that the entire area burned in the wildfire would have to be treated in 19 prescribed burns. We selected all meteorologically favorable days for conducting prescribed burns and finished all 19 counterfactual burns in about four months. However, the prescribed burning for such a large area can last several years. We ignored fuel regrowth between the burns and the postprescribed burn wildfire, which was about one year. Improvements in prescribed fire designs can be made by considering longer-term and more strategic prescribed fire treatments, such as selecting certain portions of the entire burn area. Additionally, the design of fire boundaries and the selection of meteorological conditions for prescribed fire management are also based on the ecosystem goals, aiming to protect endangered plants or animals. Incorporating ecosystem benefits into prescribed fire planning, along with simulating the evolution of fuel types and fuel loads over an extended period can be a challenge that needs to be addressed.

For smoke impact comparisons among wildfires, prescribed burns, and postprescribed burn wildfires, plume heights, emissions, and meteorological conditions were key factors affecting the exposures. Although wildfires typically have higher energy since they are more intense and have enough heat to ignite the canopy, their average plume heights were less than those of prescribed burns in this study because while prescribed fires burned only during the day, wildfires continued during the nighttime with significant drops in their plume heights. Nighttime smoke could have significantly increased local pollution levels since PBL is much lower and the air is more stagnant compared to daytime. This could have been a factor that made exposure to wildfire smoke larger than the exposures to smoke from prescribed burns and postprescribed burn wildfire. However, the population’s activity patterns also change from the daytime to the nighttime. Our study focused on outdoor air quality, but people are predominantly indoors at night. This pattern adds complexity to estimating the health impacts and trade-offs between wildfire and prescribed fire. The reduced emissions from prescribed burns and the postprescribed burn wildfire could be another factor that mitigated the exposure compared to the wildfire. The total emissions were reduced even when we assumed the occurrence of a postprescribed burn wildfire. In this study, we reduced the fuel load at the Gatlinburg wildfire region by the amount consumed in prescribed burns and assumed the postprescribed burn wildfire burned the same area as the wildfire. In reality, prescribed fire management could also reduce the burned area of a wildfire.^15^ By decreasing the fuel load, prescribed fires mitigate the heat released by fires, consequently reducing the rate of fire spread. Considering the possibility of reduced burned area in a postprescribed burn wildfire, our emissions and exposure estimates of postprescribed burn wildfire could be overestimates. However, the BlueSky-CMAQ framework has limitations on fire simulations because of the relatively coarse resolution of the fuel load data, simplified emission time profile, and parametrized plume rise process. The FCCS fuel load map incorporated in BlueSky has a 1-km resolution. The area of nonburnable (developed/barren land) region is underestimated compared to the 30-m resolution NLCD (Table S4) and has spatial discrepancies (Figure S4, S6). The emissions of prescribed burns are probably overestimated, especially for the burn on January 14, 2016. On that date, FCCS estimates the effective burned area as 1040 acres, whereas the NLCD data reports only an area of 480 acres can be burned. As for the diurnal time profile and plume rise algorithms used by BlueSky, they are simplifications of the actual processes. Wildfire emissions are expected to have different time profiles at different spreading stages. However, BlueSky assumed the burned area was equally distributed to each day during the burning periods with the same diurnal profile. Additionally, the parametrized plume height without consideration of the fire spread has limitations on following plume heights at different stages of the burn. These uncertainties and limitations in current modeling framework degrade simulation performance during the Gatlinburg wildfire periods. For Tennessee and the nearby states, the performance metrics for the daily average PM_2.5_ simulation during wildfire periods are −29.62% for NMB, 38.41% for NME, and 0.49 for R (Figure S18 and Table S5). For better estimation of burned area, consumed fuel load, and the plume height under different fire cases, coupled fire-atmosphere models such as WRF-SFIRE,^62^ QUIC-FIRE,^63^ and Wildland Urban Interface Fire Dynamics Simulator (WFDS),^64^ which simulate the fire spreading process with complex physics, are probably better suited. Some previous studies have already conducted coupled fire-atmosphere modeling to simulate the propagation of the fire and the formation of the smoke plume in wildfire and prescribed burns, and showed reasonable agreement with observations.^65,66^

Another limitation of the scenario design in the study is the assumption that prescribed burns would cover the entire Gatlinburg wildfire area. It is virtually impossible to predict the area that would burn in a wildfire; consequently, the area treated by prescribed burns would be different from the wildfire burned area. In practice, it is more likely that a smaller area would be treated by prescribed burns because of the limited resources of land management agencies. Based on an anonymous reviewer’s suggestion, we decided to evaluate the sensitivity of the air quality benefits to the extent of the treatment. For this purpose, we designed a new scenario (Scenario 3*, Text S2) where about one-half of the area burned in the Gatlinburg wildfire was treated by prescribed burns (10 out of the 19 prescribed burns from Scenario 3). We estimated the corresponding postprescribed burn wildfire emissions (Table S9), simulated the concentration impacts, and analyzed smoke person-days (Figure S24). Although the total emissions from wildland fires are similar in Scenarios 3 and 3*, the postprescribed burn wildfire, which had higher emissions in Scenario 3* compared to Scenario 3, reduced the prescribed fire benefits. Similar to Scenario 3, the prescribed burns in Scenario 3* had benefits at both low (PM_2.5_ ≤ 1 μg/m^3^) and high levels (PM_2.5_ ≥ 3 μg/m^3^) of PM_2.5_ exposures and disbenefits around 2 μg/m^3^.

The population-weighted concentration and the person-days analysis for the wildfire, prescribed fire, and postprescribed burn wildfire indicated that PM_2.5_ is a bigger concern compared to ozone and NO_2_. In this study, we found that the prescribed fires reduced the population-weighted smoke concentrations and decreased the person-days even considering the postprescribed burn wildfire period. However, the decrease is marginal and depends on several factors, including distance from the burn area and the PM threshold. The benefits of prescribed fires can be explained by several factors. First, wildfire smoke impacts a more extensive spatial range compared to prescribed fires. The wildfire with higher plume height during the daytime can be transported long distances by stronger daytime winds. The long-distance transport of smoke potentially affects urban regions with large populations. Additionally, higher emissions of wildfire lead to higher concentrations, which increases the intensity of smoke impacts and population exposure. However, prescribed fires can induce higher smoke person-days considering the postprescribed burn wildfire occurrence since the prescribed fires last longer than the wildfire, especially for the population close to the fire region. Person-days benefits from prescribed fires can be expected when the fires happen in rural regions where the population is low, and the prescribed fire prevents long-distance transported smoke effectively. Concerns may arise when the prescribed fire management is conducted at the wildland-urban interface.

In this study, since the Gatlinburg wildfire was human caused, we expected the postprescribed burn wildfire occurrence. In a large-scale study (e.g., the southeastern U.S. or CONUS), assuming the probability of postprescribed burn wildfires as either 1 or 0 may lead to varying conclusions. Besides the prevented smoke exposure or economic benefits due to the less harmful air quality impacts, the benefits from mitigating the direct wildfire damages should also be considered for policymaking since such economic loss can be a dominant factor when considering trade-offs between wildfires and prescribed fires. For instance, the direct damage caused by the Gatlinburg wildfire was 2 billion U.S. dollars.^33^ Finally, it should be remembered that there are so many other trade-off issues to be considered in making fire policy such as the impacts on vegetation, regional climate, local economies and much more.

## Data Availability

The code related to prescribed fire design, wildfire, postprescribed burn wildfire, and prescribed fire emission creation is available on GitHub: https://github.com/zli867/WFRxTradeoffs (accessed on September 1st, 2024). Other code and data related to the study will be made available on request.

## Supporting Information Available

Additional details about the methodology and results, including figures, tables, and a discussion of an additional prescribed burning scenario with half of the area treated (PDF)

## References

[ref1] Yoder J.; Engle D.; Fuhlendorf S. Liability, incentives, and prescribed fire for ecosystem management. Frontiers in Ecology and the Environment 2004, 2 (7), 361–366. 10.1890/1540-9295(2004)002[0361:LIAPFF]2.0.CO;2.

[ref2] Gleim E. R.; Zemtsova G. E.; Berghaus R. D.; Levin M. L.; Conner M.; Yabsley M. J. Frequent prescribed fires can reduce risk of tick-borne diseases. Sci. Rep. 2019, 9 (1), 9974 10.1038/s41598-019-46377-4. 31292479 PMC6620321

[ref3] Stephens S. L.; Kobziar L. N.; Collins B. M.; Davis R.; Fulé P. Z.; Gaines W.; Ganey J.; Guldin J. M.; Hessburg P. F.; Hiers K.; et al. Is fire “for the birds”? How two rare species influence fire management across the US. Frontiers in Ecology and the Environment 2019, 17 (7), 391–399. 10.1002/fee.2076.

[ref4] Fernandes P. M.; Botelho H. S. A review of prescribed burning effectiveness in fire hazard reduction. International Journal of wildland fire 2003, 12 (2), 117–128. 10.1071/WF02042.

[ref5] Wu X.; Sverdrup E.; Mastrandrea M. D.; Wara M. W.; Wager S. Low-intensity fires mitigate the risk of high-intensity wildfires in California’s forests. Science advances 2023, 9 (45), eadi4123 10.1126/sciadv.adi4123. 37948522 PMC10637742

[ref6] Fann N.; Alman B.; Broome R. A.; Morgan G. G.; Johnston F. H.; Pouliot G.; Rappold A. G. The health impacts and economic value of wildland fire episodes in the US: 2008–2012. Science of the total environment 2018, 610, 802–809. 10.1016/j.scitotenv.2017.08.024. 28826118 PMC6117838

[ref7] Afrin S. Evaluating the Impacts of Prescribed Fire on Air Quality and Public Health in the Southeastern US. Ph.D. Thesis, North Carolina State University, Raleigh, NC, USA, July 19, 2021.

[ref8] U.S. Department of Agriculture, Forest Service. Confronting the Wildfire Crisis: A Strategy for Protecting Communities and Improving Resilience; U.S. Department of Agriculture, Forest Service: Washington, DC, 2022. https://www.fs.usda.gov/sites/default/files/Wildfire-Crisis-Implementation-Plan.pdf (accessed December 19, 2024).

[ref9] Liu Y.; Prestemon J. P.; Goodrick S. L.; Holmes T. P.; Stanturf J. A.; Vose J. M.; Sun G. Future Wildfire Trends, Impacts, and Mitigation Options in the Southern United States. In Climate Change Adaptation and Mitigation Management Options: A Guide for Natural Resource Managers in Southern Forest Ecosystems; Vose J. M., Klepzig K. D., Eds.; CRC Press: Boca Raton, FL, 2014; pp 85–126.

[ref10] Jaffe D. A.; O’Neill S. M.; Larkin N. K.; Holder A. L.; Peterson D. L.; Halofsky J. E.; Rappold A. G. Wildfire and prescribed burning impacts on air quality in the United States. J. Air Waste Manage. Assoc. 2020, 70 (6), 583–615. 10.1080/10962247.2020.1749731. PMC7932990 32240055

[ref11] Williamson G.; Bowman D. M. S.; Price O. F.; Henderson S.; Johnston F. A transdisciplinary approach to understanding the health effects of wildfire and prescribed fire smoke regimes. Environmental Research Letters 2016, 11 (12), 125009 10.1088/1748-9326/11/12/125009.

[ref12] Navarro K. M.; Schweizer D.; Balmes J. R.; Cisneros R. A Review of Community Smoke Exposure from Wildfire Compared to Prescribed Fire in the United States. Atmosphere 2018, 9 (5), 185 10.3390/atmos9050185.

[ref13] Marlier M. E.; Brenner K. I.; Liu J. C.; Mickley L. J.; Raby S.; James E.; Ahmadov R.; Riden H. Exposure of agricultural workers in California to wildfire smoke under past and future climate conditions. Environmental Research Letters 2022, 17 (9), 094045 10.1088/1748-9326/ac8c58.

[ref14] Schweizer D.; Preisler H. K.; Cisneros R. Assessing relative differences in smoke exposure from prescribed, managed, and full suppression wildland fire. Air Quality, Atmosphere & Health 2019, 12, 87–95. 10.1007/s11869-018-0633-x.

[ref15] U.S. Environmental Protection Agency (EPA). Comparative Assessment of the Impacts of Prescribed Fire Versus Wildfire (CAIF): A Case Study in the Western U.S.; EPA: Research Triangle Park, NC, 2021.

[ref16] Kelp M. M.; Carroll M. C.; Liu T.; Yantosca R. M.; Hockenberry H. E.; Mickley L. J. Prescribed burns as a tool to mitigate future wildfire smoke exposure: Lessons for states and rural environmental justice communities. Earth’s Future 2023, 11 (6), e2022EF003468 10.1029/2022EF003468.

[ref17] Burke M.; Driscoll A.; Heft-Neal S.; Xue J.; Burney J.; Wara M. The changing risk and burden of wildfire in the United States. Proc. Natl. Acad. Sci. U. S. A. 2021, 118 (2), e2011048118 10.1073/pnas.2011048118. 33431571 PMC7812759

[ref18] Kramer S.; Huang S.; Mcclure C.; Chaveste M.; Lurmann F. Projected smoke impacts from increased prescribed fire activity in California’s high wildfire risk landscape. Atmos. Environ. 2023, 311, 119993 10.1016/j.atmosenv.2023.119993.

[ref19] Schweizer D.; Preisler H. K.; Cisneros R. Assessing relative differences in smoke exposure from prescribed, managed, and full suppression wildland fire. Air Quality, Atmosphere & Health 2019, 12 (1), 87–95. 10.1007/s11869-018-0633-x.

[ref20] Storey M. A.; Price O. F. Comparing the Effects of Wildfire and Hazard Reduction Burning Area on Air Quality in Sydney. Atmosphere 2023, 14 (11), 1657 10.3390/atmos14111657.

[ref21] Huang R.; Lal R.; Qin M. M.; Hu Y. T.; Russell A. G.; Odman M. T.; Afrin S.; Garcia-Menendez F.; O’Neill S. M. Application and evaluation of a low-cost PM sensor and data fusion with CMAQ simulations to quantify the impacts of prescribed burning on air quality in Southwestern Georgia, USA. J. Air Waste Manage. Assoc. 2021, 71 (7), 815–829. 10.1080/10962247.2021.1924311. 33914671

[ref22] Marlier M. E.; Brenner K. I.; Liu J. C.; Mickley L. J.; Raby S.; James E.; Ahmadov R.; Riden H. Exposure of agricultural workers in California to wildfire smoke under past and future climate conditions. Environmental Research Letters 2022, 17 (9), 094045 10.1088/1748-9326/ac8c58.

[ref23] Huang R.; Zhang X. Y.; Chan D.; Kondragunta S.; Russell A. G.; Odman M. T. Burned Area Comparisons Between Prescribed Burning Permits in Southeastern United States and Two Satellite-Derived Products. Journal of Geophysical Research-Atmospheres 2018, 123 (9), 4746–4757. 10.1029/2017JD028217.

[ref24] Val Martin M.; Honrath R.; Owen R. C.; Pfister G.; Fialho P.; Barata F. Significant enhancements of nitrogen oxides, black carbon, and ozone in the North Atlantic lower free troposphere resulting from North American boreal wildfires. Journal of Geophysical Research 2006, 111 (D23), D23S60 10.1029/2006JD007530.

[ref25] Vu B. N.; Bi J.; Wang W.; Huff A.; Kondragunta S.; Liu Y. Application of geostationary satellite and high-resolution meteorology data in estimating hourly PM2. 5 levels during the Camp Fire episode in California. Remote sensing of environment 2022, 271, 112890 10.1016/j.rse.2022.112890. 37033879 PMC10081518

[ref26] Childs M. L.; Li J. S. C.; Wen J. F.; Heft-Neal S.; Driscoll A.; Wang S. R.; Gould C. F.; Qiu M. H.; Burney J.; Burke M. Daily Local-Level Estimates of Ambient Wildfire Smoke PM2.5 for the Contiguous US. Environ. Sci. Technol. 2022, 56 (19), 13607–13621. 10.1021/acs.est.2c02934. 36134580

[ref27] Huang R.; Hu Y. T.; Russell A. G.; Mulholland J. A.; Odman M. T. The Impacts of Prescribed Fire on PM2.5 Air Quality and Human Health: Application to Asthma-Related Emergency Room Visits in Georgia, USA. International Journal of Environmental Research and Public Health 2019, 16 (13), 2312 10.3390/ijerph16132312. 31261860 PMC6651061

[ref28] Kiely L.; Neyestani S. E.; Binte-Shahid S.; York R. A.; Porter W. C.; Barsanti K. C. California Case Study of Wildfires and Prescribed Burns: PM2. 5 Emissions, Concentrations, and Implications for Human Health. Environ. Sci. Technol. 2024, 58 (12), 5210–5219. 10.1021/acs.est.3c06421. 38483184 PMC10976878

[ref29] Jones B. A.; McDermott S.; Champ P. A.; Berrens R. P. More smoke today for less smoke tomorrow? We need to better understand the public health benefits and costs of prescribed fire. International journal of wildland fire 2022, 31 (10), 918–926. 10.1071/WF22025.

[ref30] Schollaert C. L.; Jung J.; Wilkins J.; Alvarado E.; Baumgartner J.; Brun J.; Busch Isaksen T.; Lydersen J. M.; Marlier M. E.; Marshall J. D.; et al. Quantifying the smoke-related public health trade-offs of forest management. Nature Sustainability 2024, 7 (2), 130–139. 10.1038/s41893-023-01253-y.

[ref31] Scheller R. M.; Domingo J. B.; Sturtevant B. R.; Williams J. S.; Rudy A.; Gustafson E. J.; Mladenoff D. J. Design, development, and application of LANDIS-II, a spatial landscape simulation model with flexible temporal and spatial resolution. ecological modelling 2007, 201 (3–4), 409–419. 10.1016/j.ecolmodel.2006.10.009.

[ref32] ABC24 14 People Confirmed Dead In Gatlinburg Fires, 12 Identified. https://www.localmemphis.com/article/news/local/14-people-confirmed-dead-in-gatlinburg-fires-12-identified/522-b47061cc-927d-442e-ad2e-c61789f75ca5 (accessed Aug 27, 2024).

[ref33] Wikipedia 2016 Great Smoky Mountains wildfires. https://en.wikipedia.org/wiki/2016_Great_Smoky_Mountains_wildfires (accessed Oct 29).

[ref34] Commission G. F. Prescribed Burning Unit Plan. https://gatrees.org/fire-prevention-suppression/prescribed-burn/ (accessed Dec 17, 2013).

[ref35] Skamarock W. C.; Klemp J. B.; Dudhia J.; Gill D. O.; Barker D. M.; Duda M. G.; Huang X.-Y.; Wang W.; Powers J. G. A description of the advanced research WRF version 3. NCAR technical note 2008, 475, 113.

[ref36] Weir J. R.; Bidwell T. G.; Stevens R.; Mustain J. Firebreaks for prescribed burning; Oklahoma Cooperative Extension Service: 2012.

[ref37] Homer C. H.; Fry J. A.; Barnes C. A. The national land cover database. US geological survey fact sheet 2012, 3020 (4), 1–4.

[ref38] Nicholas Institute for Energy E. S. Green Firebreaks—Forest Habitats—DOI NBS Roadmap. https://nicholasinstitute.duke.edu/sites/default/files/project/nature-based-solutions-roadmap/strategy/doi-nbs-roadmap-strategy_green-firebreaks.pdf (accessed Sep 20, 2024).

[ref39] Service U. F. National Prescribed Fire Resource Mobilization Strategy. https://www.fs.usda.gov/sites/default/files/fs_media/fs_document/Rx-Fire-Strategy.pdf (accessed August 5).

[ref40] Knapp E. E.; Varner J. M.; Busse M. D.; Skinner C. N.; Shestak C. J. Behaviour and effects of prescribed fire in masticated fuelbeds. International Journal of Wildland Fire 2011, 20 (8), 932–945. 10.1071/WF10110.

[ref41] LANDFIRE. Existing Vegetation Type Layer, LANDFIRE 2.0.0; U.S. Department of the Interior, Geological Survey, and U.S. Department of Agriculture: 2016. https://landfire.gov/viewer/ (accessed May 28, 2024).

[ref42] Serra J.; Soille P. Mathematical morphology and its applications to image processing. Springer Science & Business Media: 2012; Vol. 2.

[ref43] Dijkstra E. W. A Note on Two Problems in Connexion with Graphs. In Edsger Wybe Dijkstra: His Life,Work, and Legacy; Association for Computing Machinery: 2022; Vol. 45, pp 287–290.

[ref44] Larkin N. K.; O’Neill S. M.; Solomon R.; Raffuse S.; Strand T.; Sullivan D. C.; Krull C.; Rorig M.; Peterson J.; Ferguson S. A. The BlueSky smoke modeling framework. International journal of wildland fire 2009, 18 (8), 906–920. 10.1071/WF07086.

[ref45] Ottmar R. D.; Sandberg D. V.; Riccardi C. L.; Prichard S. J. An overview of the fuel characteristic classification system—quantifying, classifying, and creating fuelbeds for resource planning. Canadian Journal of Forest Research 2007, 37 (12), 2383–2393. 10.1139/X07-077.

[ref46] Deeming J. E.; Burgan R. E.; Cohen J. D. The National Fire-Danger Rating System - 1978; Gen. Tech. Rep. INT-GTR-39; U.S. Department of Agriculture, Forest Service, Intermountain Forest and Range Experiment Station: Ogden, UT, 1977; 63 p.

[ref47] Ottmar R. D.; Burns M. F.; Hall J. N.; Hanson A. D. CONSUME: Users Guide; Gen. Tech. Rep. PNW-GTR-304; U.S. Department of Agriculture, Forest Service, Pacific Northwest Research Station: Portland, OR, 1993.

[ref48] Prichard S. J.; O’Neill S. M.; Eagle P.; Andreu A. G.; Drye B.; Dubowy J.; Urbanski S.; Strand T. M. Wildland fire emission factors in North America: synthesis of existing data, measurement needs and management applications. International Journal of Wildland Fire 2020, 29 (2), 132–147. 10.1071/WF19066.

[ref49] Feng X.; Mickley L. J.; Bell M. L.; Liu T.; Fisher J. A.; Val Martin M. Improved estimates of smoke exposure during Australia fire seasons: importance of quantifying plume injection heights. Atmos. Chem. Phys. 2024, 24 (5), 2985–3007. 10.5194/acp-24-2985-2024.

[ref50] Li Y.; Tong D.; Ma S.; Freitas S. R.; Ahmadov R.; Sofiev M.; Zhang X.; Kondragunta S.; Kahn R.; Tang Y.; Baker B.; Campbell P.; Saylor R.; Grell G.; Li F. Impacts of estimated plume rise on PM2.5 exceedance prediction during extreme wildfire events: A comparison of three schemes (Briggs, Freitas, and Sofiev). Atmos. Chem. Phys. 2023, 23, 3083 10.5194/acp-23-3083-2023.

[ref51] Briggs G. A. Plume rise and buoyancy effects. Atmospheric science and power production 1984, 327, 366.

[ref52] Byun D.; Schere K. L. Review of the governing equations, computational algorithms, and other components of the Models-3 Community Multiscale Air Quality (CMAQ) modeling system. Applied mechanics reviews 2006, 59 (2), 51–77. 10.1115/1.2128636.

[ref53] (accessed 2023, April 13)

[ref54] Fehsenfeld F.; Calvert J.; Fall R.; Goldan P.; Guenther A. B.; Hewitt C. N.; Lamb B.; Liu S.; Trainer M.; Westberg H.; et al. Emissions of volatile organic compounds from vegetation and the implications for atmospheric chemistry. Global biogeochemical cycles 1992, 6 (4), 389–430. 10.1029/92GB02125.

[ref55] (accessed 2024, March 28)

[ref56] WorldPop. Global High Resolution Population Denominators Project. Center for International Earth Science Information Network (CIESIN), University of Southampton: Southampton, UK, 2018.https://dx.doi.org/10.5258/SOTON/WP00670 (accessed Aug 27, 2024).

[ref57] Alexander L. K.; Lopes B.; Ricchetti-Masterson K; Yeatts K. B. Calculating Person-Time. https://sph.unc.edu/wp-content/uploads/sites/112/2015/07/nciph_ERIC4.pdf (accessed May 22, 2024)..

[ref58] Weisstein E. W. Heaviside step function. https://mathworld.wolfram.com/HeavisideStepFunction.html (accessed Dec 19, 2024).

[ref59] USDA Forest Service AirFire Research Team. FCCS2 Fuel Map Data. https://github.com/pnwairfire/fccsmap/blob/master/fccsmap/data/fccs2_fuelload.nc (accessed Aug 27, 2024).

[ref60] Emery C.; Liu Z.; Russell A. G.; Odman M. T.; Yarwood G.; Kumar N. Recommendations on statistics and benchmarks to assess photochemical model performance. J. Air Waste Manage. Assoc. 2017, 67 (5), 582–598. 10.1080/10962247.2016.1265027. 27960634

[ref61] Appel K. W.; Napelenok S. L.; Foley K. M.; Pye H. O.; Hogrefe C.; Luecken D. J.; Bash J. O.; Roselle S. J.; Pleim J. E.; Foroutan H.; et al. Description and evaluation of the Community Multiscale Air Quality (CMAQ) modeling system version 5.1. Geoscientific model development 2017, 10 (4), 1703–1732. 10.5194/gmd-10-1703-2017. 30147852 PMC6104654

[ref62] Mandel J.; Beezley J. D.; Kochanski A. K. Coupled atmosphere-wildland fire modeling with WRF 3.3 and SFIRE 2011. Geoscientific Model Development 2011, 4 (3), 591–610. 10.5194/gmd-4-591-2011.

[ref63] Linn R. R.; Goodrick S. L.; Brambilla S.; Brown M. J.; Middleton R. S.; O’Brien J. J.; Hiers J. K. QUIC-fire: A fast-running simulation tool for prescribed fire planning. Environmental Modelling & Software 2020, 125, 104616 10.1016/j.envsoft.2019.104616.

[ref64] McGrattan K.; Hostikka S.; McDermott R.; Floyd J.; Weinschenk C.; Overholt K. Fire dynamics simulator user’s guide. NIST special publication 2013, 1019 (6), 1–339.

[ref65] Shamsaei K.; Juliano T. W.; Roberts M.; Ebrahimian H.; Kosovic B.; Lareau N. P.; Taciroglu E. Coupled fire-atmosphere simulation of the 2018 Camp Fire using WRF-Fire. International journal of wildland fire 2023, 32 (2), 195–221. 10.1071/WF22013.

[ref66] Kochanski A. K.; Jenkins M. A.; Mandel J.; Beezley J. D.; Clements C. B.; Krueger S. Evaluation of WRF-SFIRE performance with field observations from the FireFlux experiment. Geoscientific Model Development 2013, 6 (4), 1109–1126. 10.5194/gmd-6-1109-2013.

