# Supplementary material for: The Trade-offs between Wildfires and Prescribed Fires: A Case Study for 2016 Gatlinburg Wildfires
Source: ACS EST Air. 2025 Jan 9;2(2):236–48. doi: 10.1021/acsestair.4c00233 (PMC11833759; doi:10.1021/acsestair.4c00233)
Supplement: Supplementary file 1 — ea4c00233_si_001.pdf [file ea4c00233_si_001.pdf]

Supplemental Material for

The Tradeoffs Between Wildfires and Prescribed Fires: A Case Study for 2016 Gatlinburg Wildfires

Zongrun Li[1], Ambarish Vaidyanathan[1,2], Kamal J. Maji[1], Yongtao Hu[1], Susan M. O'Neill[3], Armistead G. Russell[1], M. Talat Odman[1, *]

[1] School of Civil and Environmental Engineering, Georgia Institute of Technology, Atlanta, GA 30332, USA

[2] National Center for Environmental Health, Centers for Disease Control and Prevention, Atlanta, GA 30341, USA

[3] United States Department of Agriculture Forest Service, Pacific Northwest Research Station, Seattle, WA 98103, USA

[*]Corresponding author: M. Talat Odman (odman@gatech.edu)

This material includes 44 pages, 24 figures, 9 tables.

Table of Contents

Figure S1. The left figure shows the area burned in the Gatlinburg wildfire and the nearby 12-km resolution WRF grid cells. The blue star indicates the WRF grid cell which provides the meteorological data. The right figure is the map of the wildfire region and its nearby communities. The Great Smoky Mountain region and Pigeon Forge City are indicated. The red, blue, brown, and purple lines show the boundary of Sevier, Swain, Jackson County, and Greenville counties. Black lines show the boundaries of states. TN: Tennessee; NC: North Carolina; SC: South Carolina; GA: Georgia...6

Figure S2. Land cover type of the area burned in the Gatlinburg wildfire according to 2016 NLCD data. 7

Figure S3. Terrain slope of the area burned in the Gatlinburg wildfire according to LANDFIRE topographic data. 8

Algorithm S1. Extract Boundary Line in Raster Data 9

Table S1. BlueSky Framework Configuration...10

Figure S4. Land cover type of the area burned in the Gatlinburg wildfire according to 2016 NLCD data aligned with designed prescribed fire boundaries (red lines). 11

Figure S5. Terrain slope in the area burned in Gatlinburg wildfire according to LANDFIRE topographic data aligned with designed prescribed fire boundaries (red lines). 12

Table S2. Emissions (unit: metric tons) under the wildfire, prescribed burns, and post-prescribed burn wildfire cases for the Gatlinburg wildfire region. 13

Figure S6. FCCS (version 2) fuel map in Gatlinburg region. The red lines show the Gatlinburg counterfactual prescribed burns boundaries. 14

Figure S7. Total emissions (in metric tons) of NO_x, $PM_{2.5}$, and VOC for each designed prescribed burn. 15

Figure S8. Prescribed fire emission diurnal time profile. The ratio is the percentage of hourly emission over the total daily emission...16

Figure S9. Wildfire emission diurnal time profile. The ratio is the percentage of hourly emission over the total daily emission...17

Figure S10. Hourly plume heights under the prescribed burns, wildfire, and post-prescribed burn wildfire cases. The purple dashed line indicates the mean values of the plume heights in each case. Each point indicates an hourly plume height. The maximum, minimum, and mean plume heights are 3294.7, 1248.7, 2099.0 m for the prescribed fire case; 6338.3, 280.6, 1854.1 m for the wildfire case; 4188.9, 191.3, 1250.6 m for the post-prescribed burn wildfire case. 18

Figure S11. Re-gridded population in the southeastern United States. The black boundaries show the southeastern states focused in this study...19

Text S1. CMAQ Performance Evaluation 20

Table S3. Daily averaged $PM_{2.5}$, MDA8-O_3, and 1-hr max NO_2 performance during the study, i.e., the burn dates of counterfactual prescribed fires and their following two days, and November

25th, 2016 to December 1st, 2016, which covers the Gatlinburg wildfire and the following two days. 20

Figure S12. County-level population-weighted $PM_{2.5}$, MDA8-O_3, and 1-hr max NO_2 concentrations due to smoke impacts during the wildfire, post-prescribed burn wildfire, and prescribed burn periods. 21

Figure S13. The spatial distributions of prescribed fire prevented population-weighted exposures (WF conc– Rx conc – post-Rx WF conc) of $PM_{2.5}$, MDA8-O_3, and NO_2. 22

Figure S14. The prescribed fire prevented person-days (unit: million person-day) under different spatial ranges for a certain burn impact concentration threshold. The dashed line shows zero. The sum of prescribed fire and post-prescribed burn wildfire has lower person-days than wildfire when the line is above the dashed line. EUS: Eastern U.S. (the entire study domain; same as the black line in Figure 5); TN-NC: Tennessee and North Carolina; GA: Georgia. 23

Figure S15. The relative prescribed fire prevented person-days compared to wildfire person-time under different spatial ranges for a certain burn impact concentration threshold. The dashed line shows zero. The relative reduced person days is calculated by:$(\boldsymbol{PDWF - PDRx - PDpost - Rx\,WF})\boldsymbol{PDWF}$, where PD is person-days. EUS: Eastern U.S. (the entire study domain); TN-NC: Tennessee and North Carolina; GA: Georgia. 24

Figure S16. Grid-based prescribed fire boundary design. The prescribed fire boundary is the intersection between a rectangular grid and the Gatlinburg wildfire boundary. The burn dates are the same as the prescribed fire design with fire break considerations. However, the burned area for each date is different due to the different fire boundaries. 25

Figure S17. The mean difference of daily average $PM_{2.5}$ between grid-based design and fire break-based design during the prescribed fire burn dates. The blue tones show places where the grid-based design leads to higher $PM_{2.5}$ concentrations than the fire break-based design. The red tones show places where the grid-based design leads to lower $PM_{2.5}$ concentrations than the fire break-based design. 26

Table S4. Designed prescribed burns region area, the non-burnable (developed/barren land) area, and the effective burned area in FCCS and NLCD datasets (unit: acres). 27

Table S5. Daily averaged $PM_{2.5}$, MDA8-O_3, and 1-hr max NO_2 performance during the study, i.e., November 25th, 2016 to December 1st, 2016 in Alabama, Georgia, Kentucky, Virginia, North Carolina, South Carolina, and Tennessee., which covers the Gatlinburg wildfire and the following two days. 28

Figure S18. Model evaluation by comparing simulations to observations for daily average $PM_{2.5}$, MDA8-O_3, and 1-hr max NO_2 (the wildfire burn dates and the following two days, Nov 25th, 2016 to Dec 1st, 2016) in Alabama, Georgia, Kentucky, Virginia, North Carolina, South Carolina, and Tennessee. The spatial plots on the left show the Pearson correlation coefficient (also known as R) value between simulation and observation for each monitor in the study domain. The density scatterplots on the right show the relationship between all observations and simulations. The black dashed line is the unity (1:1) slope line. The red line shows the linear relationship between simulation and observations. The R^2 performance and 95% confidential interval of slopes and intercepts of the regression line are indicated. N shows the total number of data points in the linear regression........29

Table S6. Model performance for daily averaged $PM_{2.5}$, MDA8-O_3, and 1-hr max NO_2 in Scenario 2 during the designed counterfactual prescribed burn days and the two days following each burn (for a total of 45 days between December 3rd 2015 and March 29th, 2016 as listed in Table S8), in the focused states (shown by the red boundaries in Figure 1). 31

Figure S19. Model evaluation by comparing Scenario 2 simulation results to observations for daily average $PM_{2.5}$, MDA8-O_3, and 1-hr max NO_2 on counterfactual prescribed burn days and the following two days. The spatial plots on the left show the Pearson correlation coefficient value at each monitor in the study domain. The density scatterplots on the right show the relationship between observations and simulation results. The black dashed line is the unity (1:1) slope line. The red line is the linear regression line whose equation is shown along with the 95% confidence interval for the slope and intercept. R^2 is the coefficient of determination and N is the number of data points in the linear regression. 32

Table S7. Model performance for daily averaged $PM_{2.5}$, MDA8-O_3, and 1-hr max NO_2 in Scenario 2 during the Gatlinburg wildfire period and the following two days (for a total of 7 days between November 25th, 2016 and December 1st, 2016) in the focused states (shown by the red boundaries in Figure 1). 33

Figure S20. Model evaluation by comparing Scenario 2 simulation results to observations for daily average $PM_{2.5}$, MDA8-O_3, and 1-hr max NO_2 on the wildfire days and the following two days. The spatial plots on the left show the Pearson correlation coefficient value at each monitor in the study domain. The density scatterplots on the right show the relationship between observations and simulation results. The black dashed line is the unity (1:1) slope line. The red line is the linear regression line whose equation is shown along with the 95% confidence interval for the slope and intercept. R^2 is the coefficient of determination and N is the number of data points in the linear regression. 34

Figure S21. Mean daily average $PM_{2.5}$, MDA8-O_3, and 1-hr max NO_2 of smoke impacts during the wildfire, post-prescribed burn wildfire, and prescribed burns periods. The smoke impacts are calculated by subtracting the baseline scenario concentrations from fire cases. The impacts are plotted using the same symmetrical log scale for all three types of fires.Table S8. Burn dates, CMAQ simulation and study focused time periods for different fire cases. 35

Figure S22. The person-days under prescribed fire (Rx: blue), wildfire (WF: red), and post-prescribed burn wildfire (Post-Rx WF: orange) cases, for burn impact concentration threshold from 1 to 15 $\mu g/m^3$ at 1 $\mu g/m^3$ intervals. The y-axis is in symmetrical log scale. The green bars show the sum of person-days from Rx and Post-Rx WF. The black bars show the person-days prevented by prescribed fires, calculated as the difference between person-days of wildfire exposure and the combined person-days of prescribed fire and post-prescribed burn wildfire exposure. 37

Text S2. Scenario 3* 38

Scenario 3* design: 38

Figure S23. Blue line indicates the part of the Gatlinburg wildfire area treated with prescribed burns in Scenario 3*. Each prescribed burn's boundary is filled with a different color. The first line of text in each boundary indicates the burn date (year-month-day in YYMMDD format), and the second line indicates the burned area (acres). Red line shows the boundary of the part without prescribed burn treatment. 39

Emissions: 39

Table S9. Wildland fire emissions (unit: metric tons) under different scenarios. 39

Smoke Person-days Analysis: 40

Figure S24. The person-days under Gatlinburg wildfire (WF) and prescribed fire (Rx*) and post-prescribed burn wildfire (Post-Rx WF*) cases of Scenario 3* for specific burn impact concentration thresholds, represented by red, blue, and orange lines, respectively. The green line shows the sum of person-days for Scenario 3*. The black line shows the person-days prevented by prescribed burns Rx*, calculated as the difference in person-days of exposure between Gatlinburg wildfire and Scenario 3*. The dashed line represents zero person-days. 41

Disclaimer 42

Reference: 43

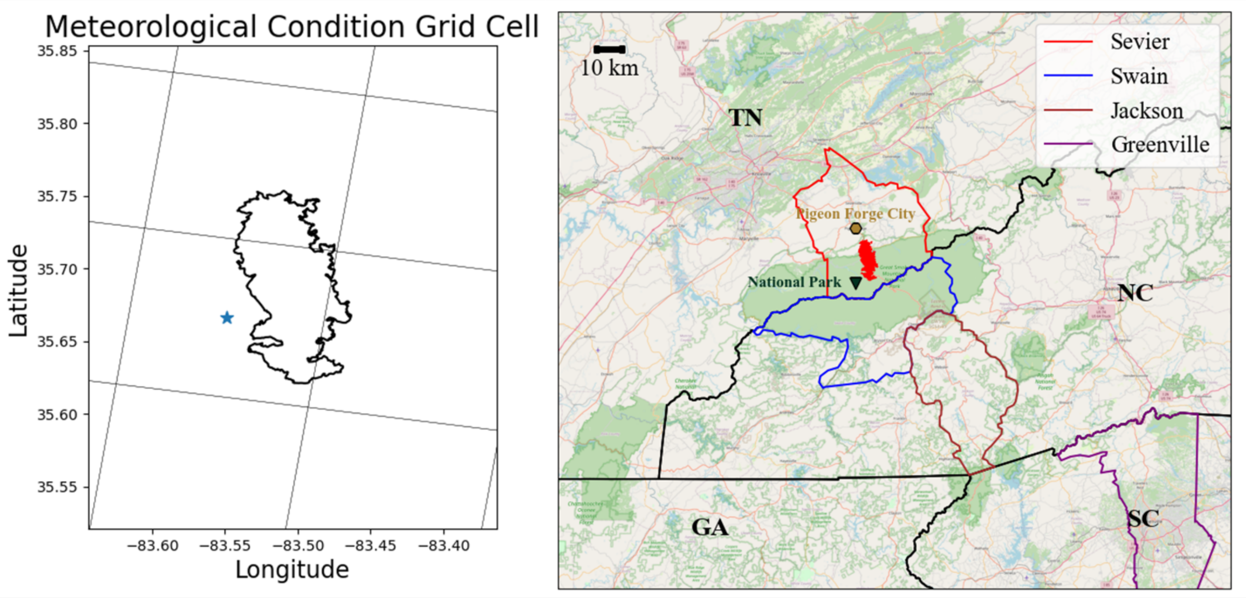

Figure S1. The left figure shows the area burned in the Gatlinburg wildfire and the nearby 12-km resolution WRF grid cells. The blue star indicates the WRF grid cell which provides the meteorological data. The right figure is the map of the wildfire region and its nearby communities. The Great Smoky Mountain region and Pigeon Forge City are indicated. The red, blue, brown, and purple lines show the boundary of Sevier, Swain, Jackson County, and Greenville counties. Black lines show the boundaries of states. TN: Tennessee; NC: North Carolina; SC: South Carolina; GA: Georgia.

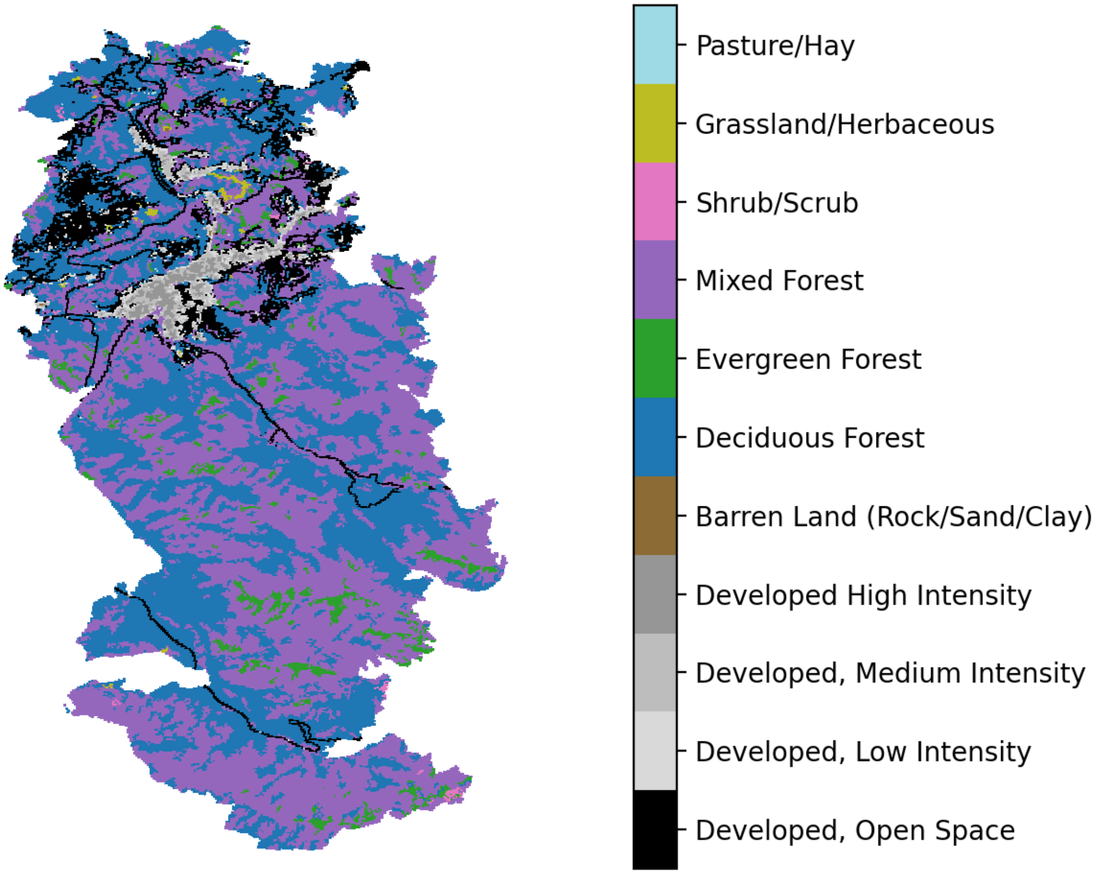

Figure S2. Land cover type of the area burned in the Gatlinburg wildfire according to 2016 NLCD data.

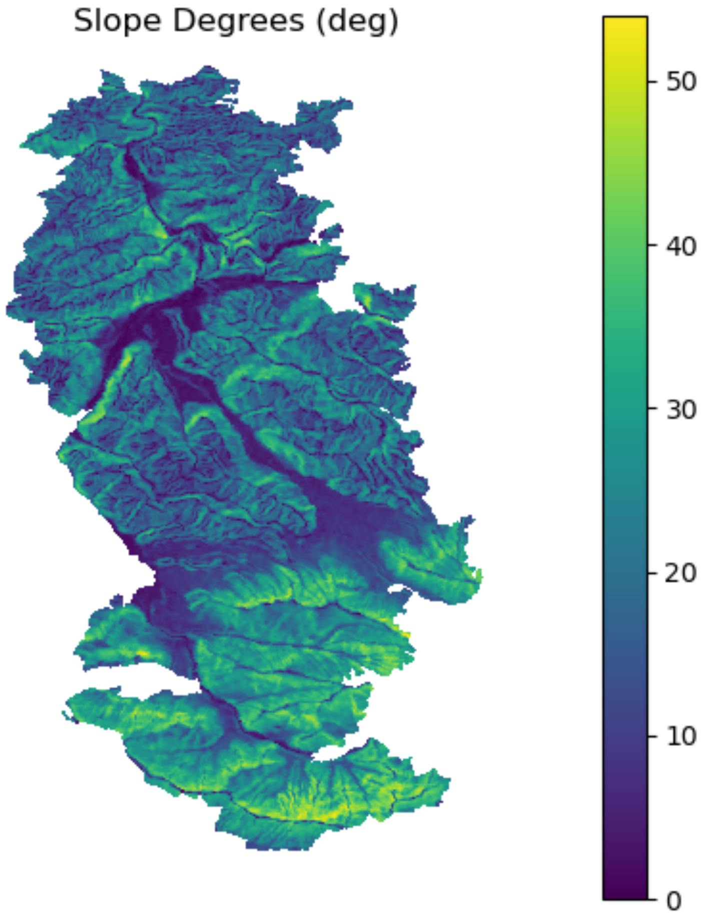

Figure S3. Terrain slope of the area burned in the Gatlinburg wildfire according to LANDFIRE topographic data.

Algorithm S1. Extract Boundary Line in Raster Data

Algorithm 1 Extract Boundary Line in Raster Data

Input: Raster data; target values in raster data for boundary design; start and end points of the boundary line
Output: Boundary line

1: Generate a mask matrix $\mathcal{M}$ based on raster data and target values for boundary design: set the grid cell value to 1 when the grid cell value in raster data is equal to target values. Otherwise, set the grid cell value to 0.
2: Conduct dilation operations to simplify the mask matrix $\mathcal{M}$. When a block in the mask matrix is all 1s, the dilation operation extracts the boundary of the block and sets the values inside of that block to 0. The simplified mask matrix is $\mathcal{M}'$.
3: Using the user-selected start ($\mathcal{S}$) and end points ($\mathcal{E}$) find the nearest corresponding grid cells ($\mathcal{S}'$ and $\mathcal{E}'$, separately) with value 1 in $\mathcal{M}'$. These two points are the start and end points of the path we will determine following the target values.
4: For a certain grid cell, the connected grid cells are defined by the Moore neighborhoods whose values in $\mathcal{M}'$ are 1. The distance between connected grid cells and the grid cell is assumed to be 1. We used a uniform-cost search algorithm to find a connected path between $\mathcal{S}'$ and $\mathcal{E}'$ in mask matrix $\mathcal{M}'$.

Table S1. BlueSky Framework Configuration

Data	Models (Settings)
Meteorological data	North American Mesoscale Forecast System 12km (NAM) [1]
Fuel type	Fuel Characteristic Classification System version 2 (FCCS) [2]
Fuel moisture	National Fire Danger Rating System (NFDRS) [3]
Fuel consumption	CONSUME model [4]
Emissions	O'Neill-Prichard emission factors [5]
Plume rise	Fire Emission Production Simulator (FEPS) with Briggs plume top behaviors [6]

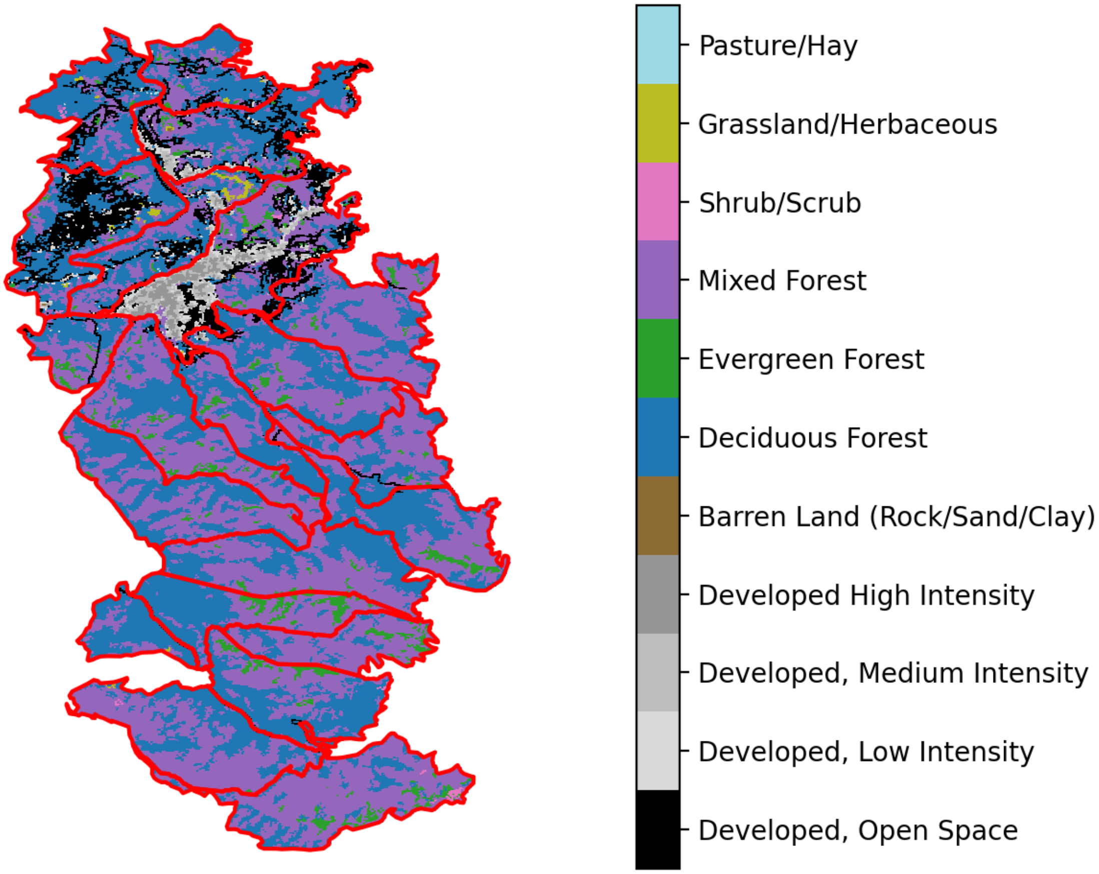

Figure S4. Land cover type of the area burned in the Gatlinburg wildfire according to 2016 NLCD data aligned with designed prescribed fire boundaries (red lines).

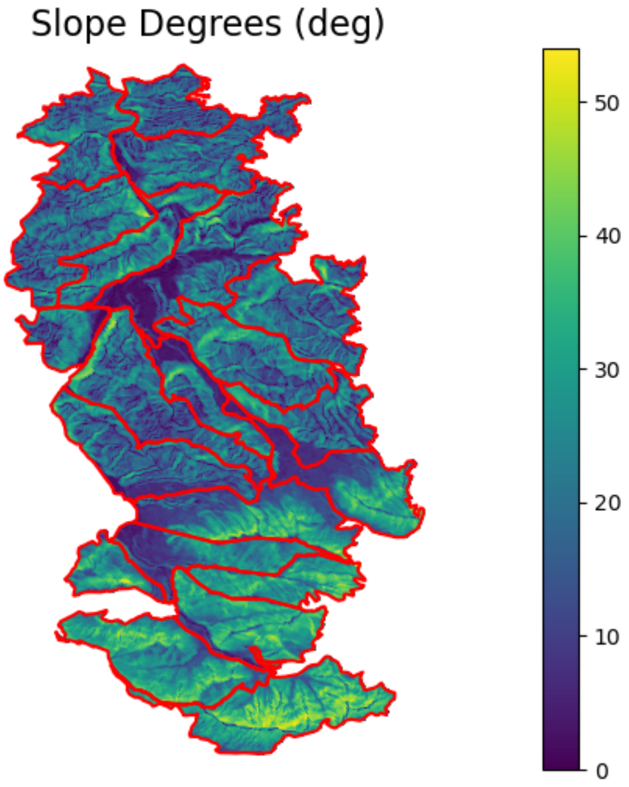

Figure S5. Terrain slope in the area burned in Gatlinburg wildfire according to LANDFIRE topographic data aligned with designed prescribed fire boundaries (red lines).

Table S2. Emissions (unit: metric tons) under the wildfire, prescribed burns, and post-prescribed burn wildfire cases for the Gatlinburg wildfire region.

Fire type	NO_x	$PM_{2.5}$	VOC
Wildfire	736.3	5609.4	6307.6
Rx burn	359.9	2914.3	3322.1
Post-Rx WF	301.0	2363.0	2697.8

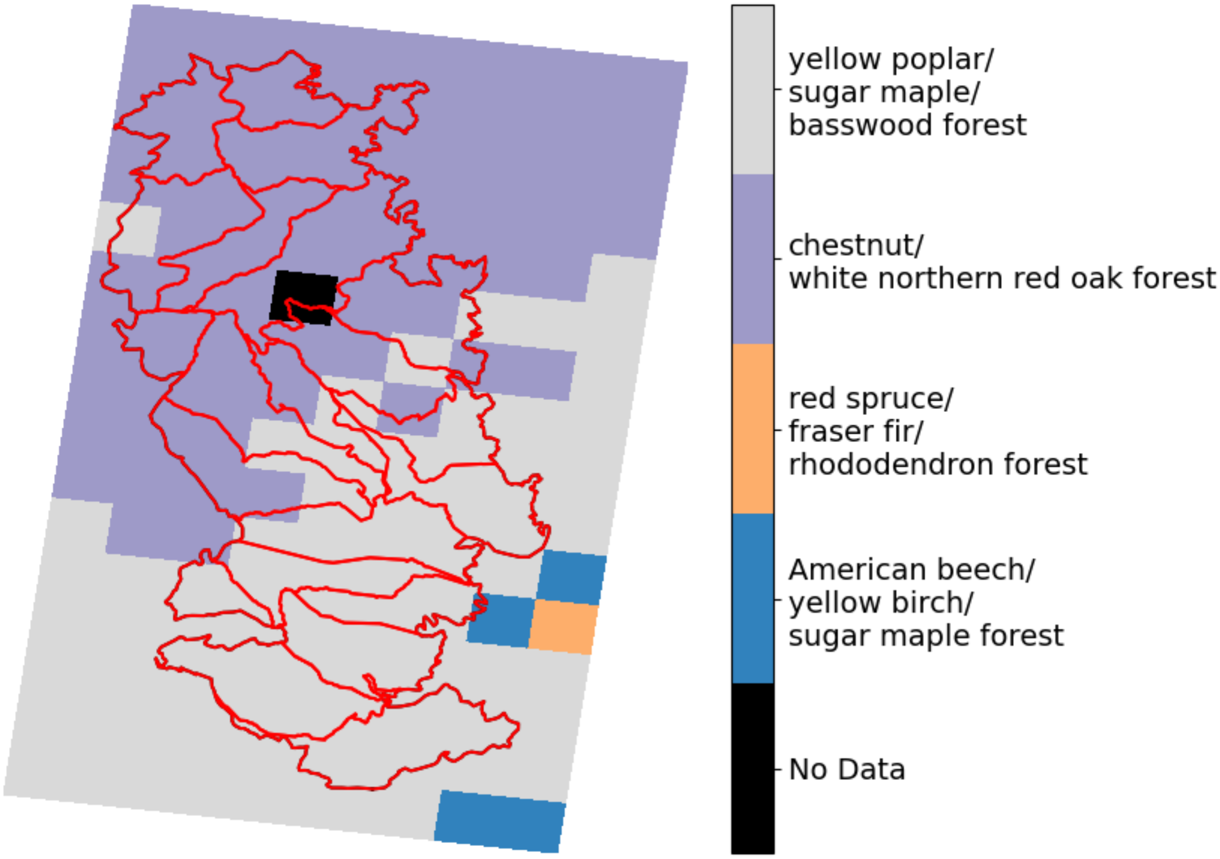

Figure S6. FCCS (version 2) fuel map in Gatlinburg region. The red lines show the Gatlinburg counterfactual prescribed burns boundaries.

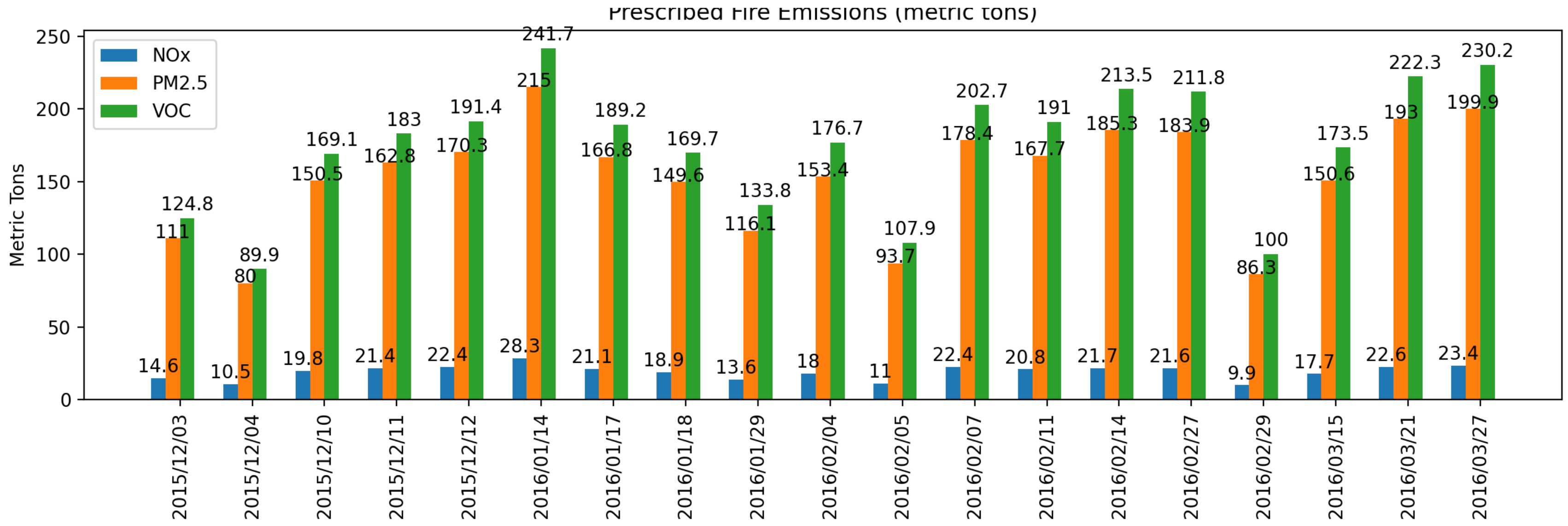

Figure S7. Total emissions (in metric tons) of NO_x, $PM_{2.5}$, and VOC for each designed prescribed burn.

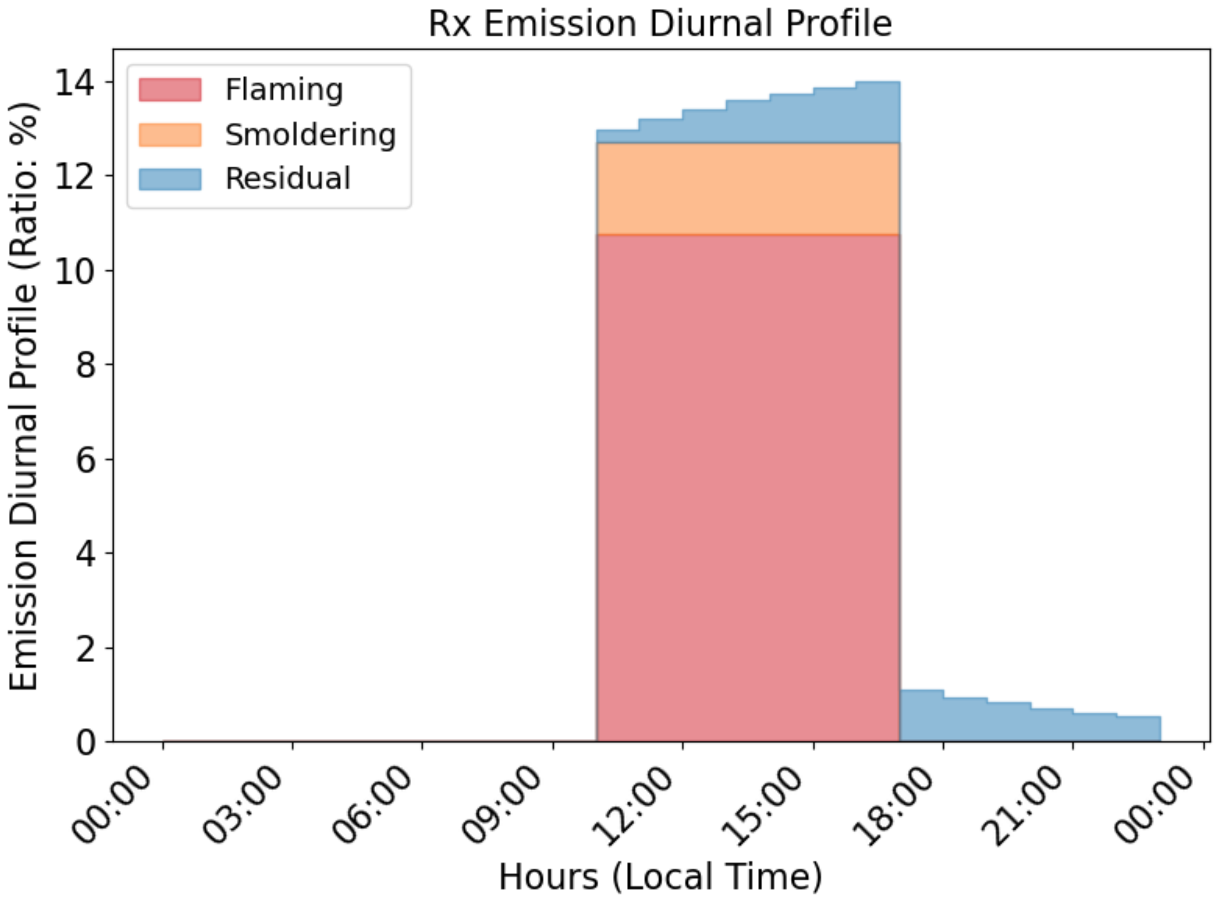

Figure S8. Prescribed fire emission diurnal time profile. The ratio is the percentage of hourly emission over the total daily emission.

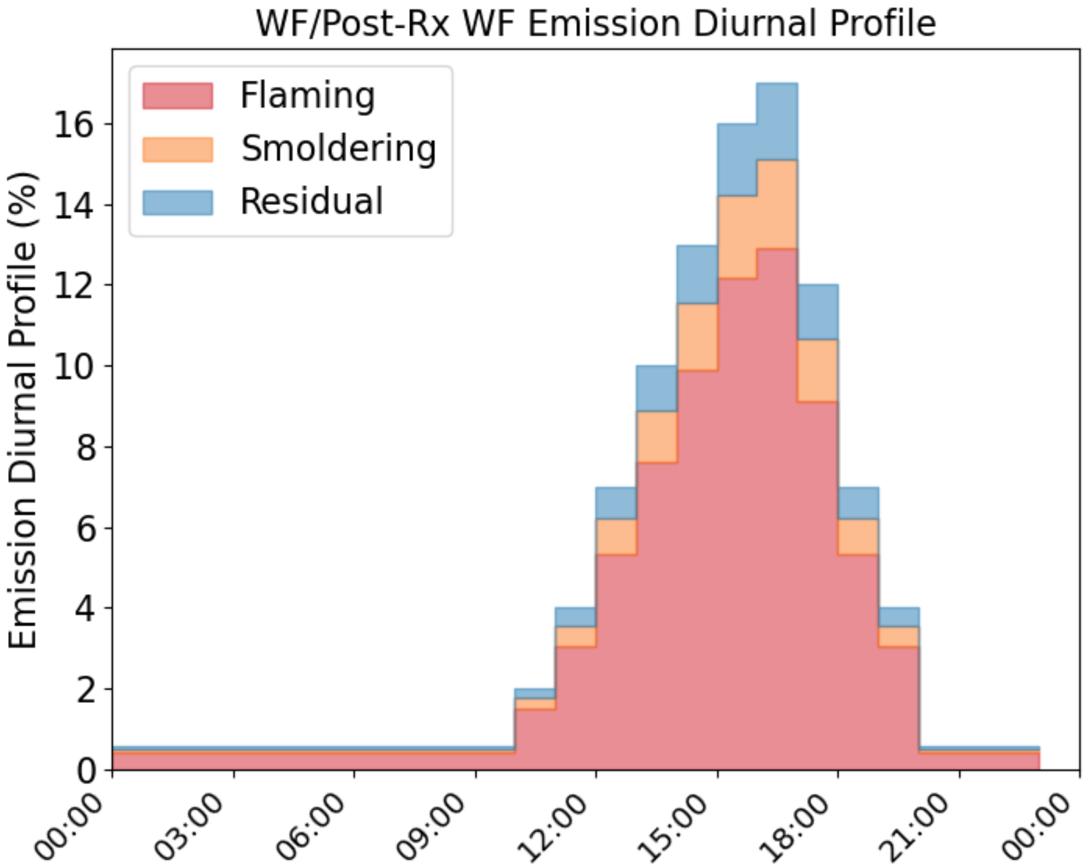

Figure S9. Wildfire emission diurnal time profile. The ratio is the percentage of hourly emission over the total daily emission.

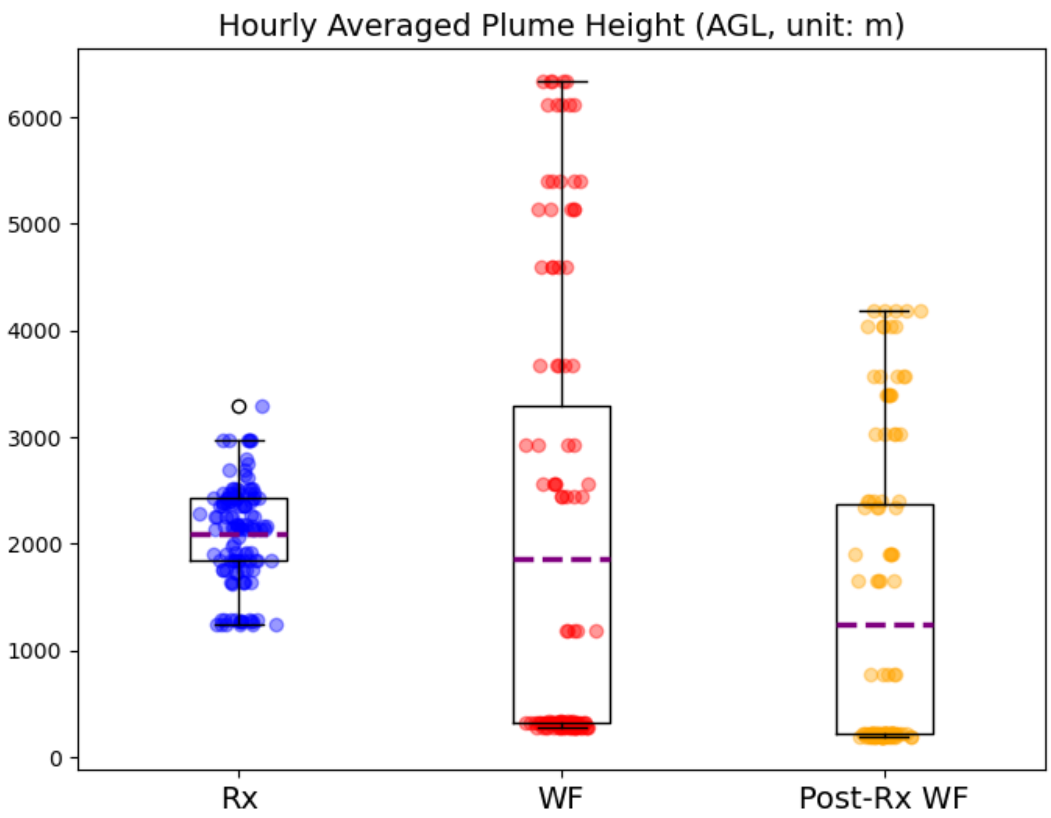

Figure S10. Hourly plume heights under the prescribed burns, wildfire, and post-prescribed burn wildfire cases. The purple dashed line indicates the mean values of the plume heights in each case. Each point indicates an hourly plume height. The maximum, minimum, and mean plume heights are 3294.7, 1248.7, 2099.0 m for the prescribed fire case; 6338.3, 280.6, 1854.1 m for the wildfire case; 4188.9, 191.3, 1250.6 m for the post-prescribed burn wildfire case.

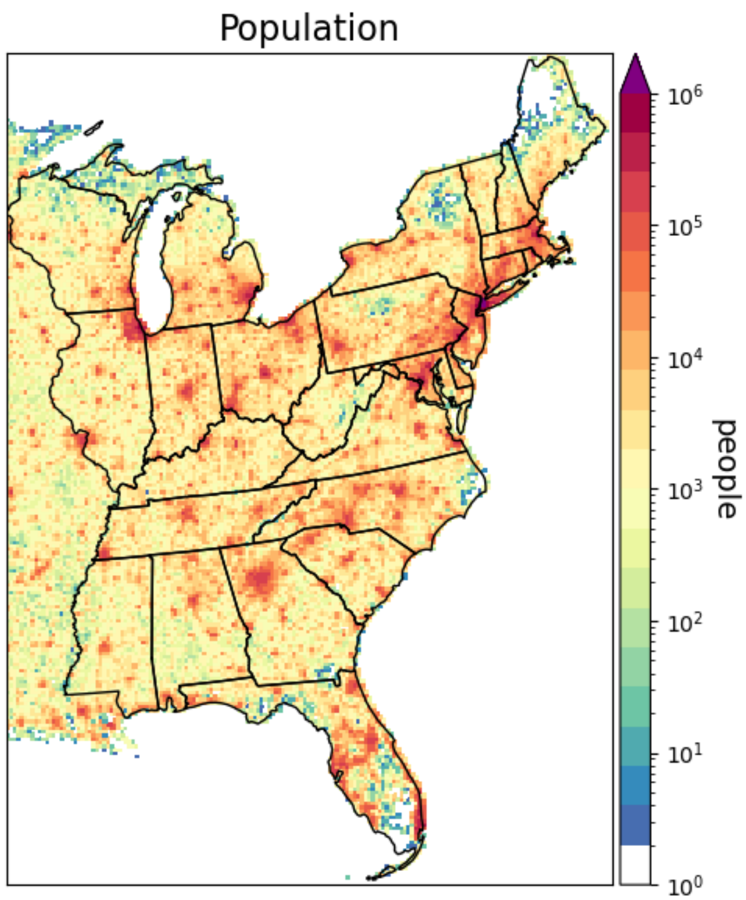

Figure S11. Re-gridded population in the southeastern United States. The black boundaries show the southeastern states focused in this study.

Text S1. CMAQ Performance Evaluation

Statistical measures for model performance evaluation [7]:

Mean bias (MB) = $\frac{1}{N}\sum(M_j - O_j)$

Mean error (ME) = $\frac{1}{N}\sum|M_j - O_j|$

Root mean square error (RMSE) = $\sqrt{\frac{1}{N}\sum(M_j - O_j)^2}$

Centered RMSE (CRMSE) = $\sqrt{\frac{1}{N}\sum[(M_j - \overline{M_j}) - (O_j - \bar{O}_j)]^2}$

Normalized mean bias (NMB) = $\frac{\sum(M_j - O_j)}{\sum O_j} \times 100$

Normalized mean error (NME) = $\frac{\sum|M_j - O_j|}{\sum O_j} \times 100$

Mean normalized bias (MNB) = $\frac{1}{N}\sum\frac{(M_j - O_j)}{O_j} \times 100$

Mean normalized error (MNE) = $\frac{1}{N}\sum\frac{|M_j - O_j|}{O_j} \times 100$

Fractional bias (FB) = $\frac{2}{N}\sum\frac{(M_j - O_j)}{(M_j + O_j)} \times 100$

Fractional error (FE) = $\frac{2}{N}\sum\frac{|M_j - O_j|}{|M_j + O_j|} \times 100$

Index of agreement (IOA) = $1 - \frac{\sum(M_j - O_j)^2}{\sum(|M_j - \bar{M}_j| + |O_j - \bar{O}_j|)^2}$

Correlation coefficient (Pearson R) = $\frac{\sum|M_j - \bar{M}_j| \times |O_j - \bar{O}_j|}{\sqrt{\sum(M_j - \bar{M}_j)^2 \times \sum(O_j - \bar{O}_j)^2}}$

Table S3. Daily averaged $PM_{2.5}$, MDA8-O_3, and 1-hr max NO_2 performance during the study, i.e., the burn dates of counterfactual prescribed fires and their following two days, and November 25th, 2016 to December 1st, 2016, which covers the Gatlinburg wildfire and the following two days.

	**Daily averaged $PM_{2.5}$**	**MDA8-O_3**	**1-hr max NO_2**
MB	-0.15	-1.99	-4.55
ME	3.07	5.00	8.29
RMSE	4.59	6.43	11.13
CRMSE	4.59	6.11	10.15
NMB	-1.85	-5.69	-21.24
NME	36.78	14.33	38.70
MNB	14.42	1.61	-5.93
MNE	48.29	19.07	46.06
FB	-5.76	-3.26	-23.41
FE	38.05	16.10	48.31
IOA	0.79	0.85	0.77
Pearson R	0.65	0.81	0.63

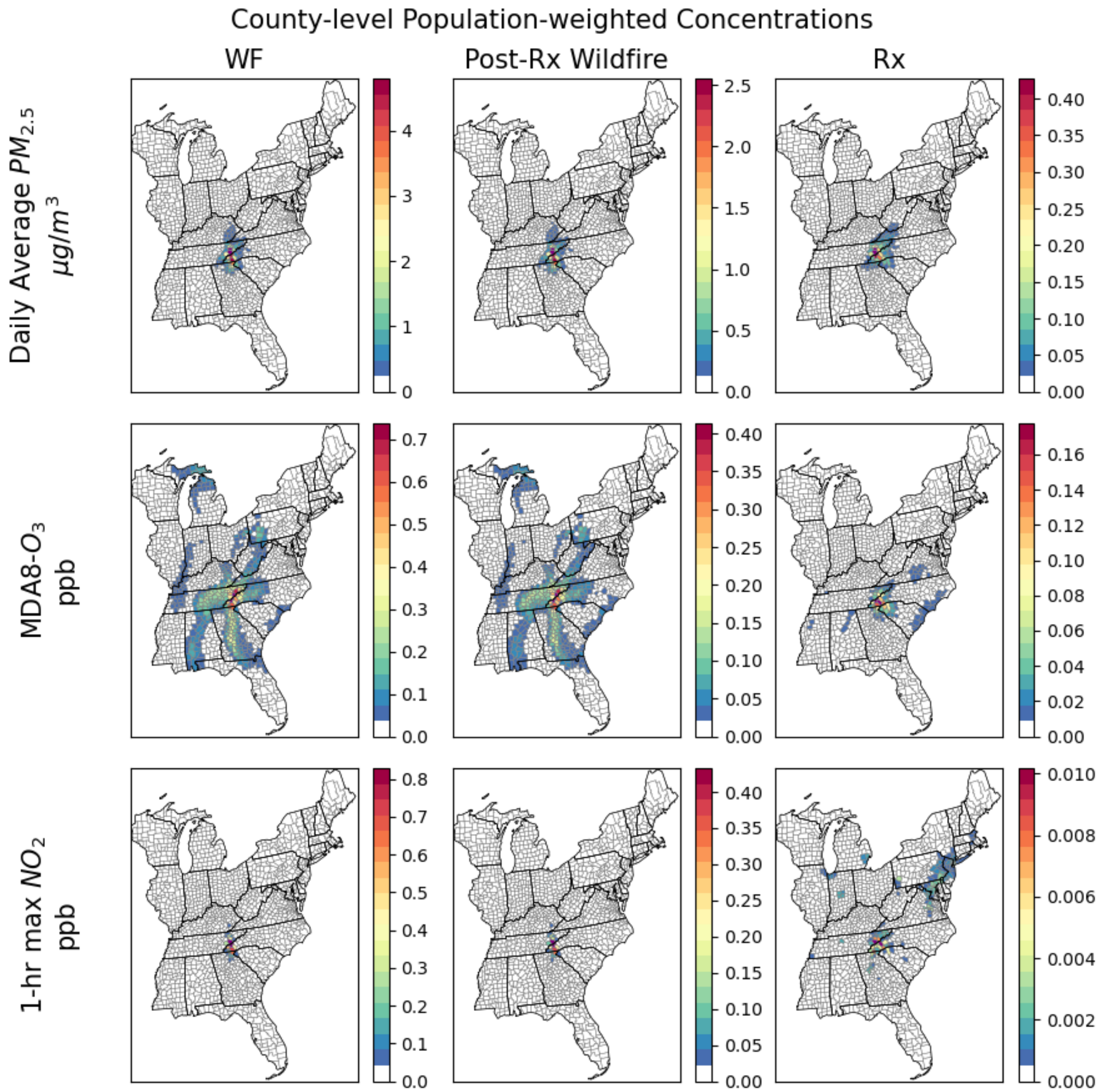

Figure S12. County-level population-weighted $PM_{2.5}$, MDA8-O_3, and 1-hr max NO_2 concentrations due to smoke impacts during the wildfire, post-prescribed burn wildfire, and prescribed burn periods.

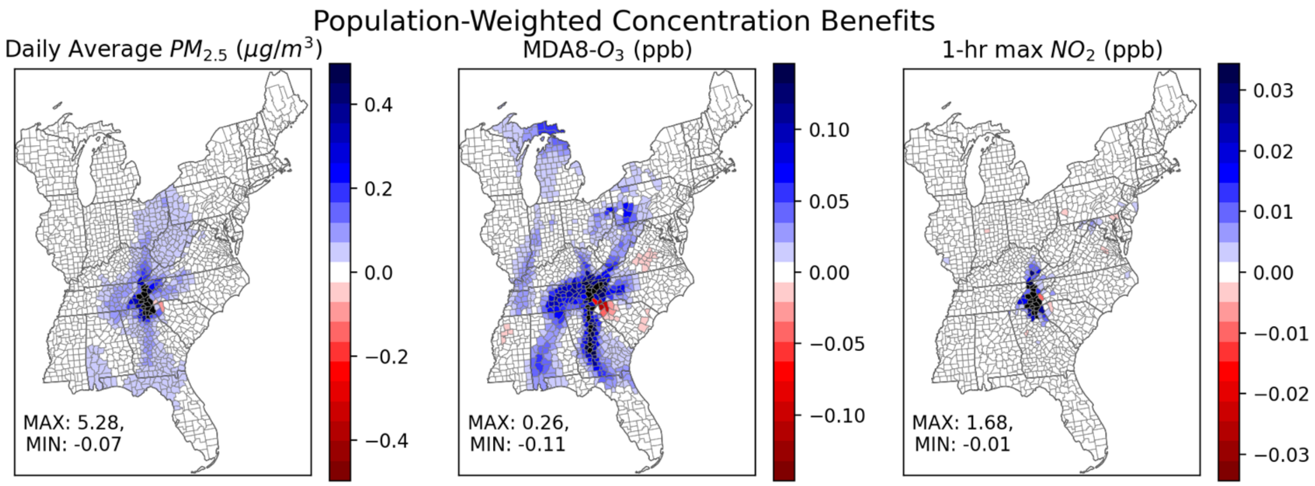

Figure S13. The spatial distributions of prescribed fire prevented population-weighted exposures (WF conc– Rx conc – post-Rx WF conc) of $PM_{2.5}$, MDA8-O_3, and NO_2.

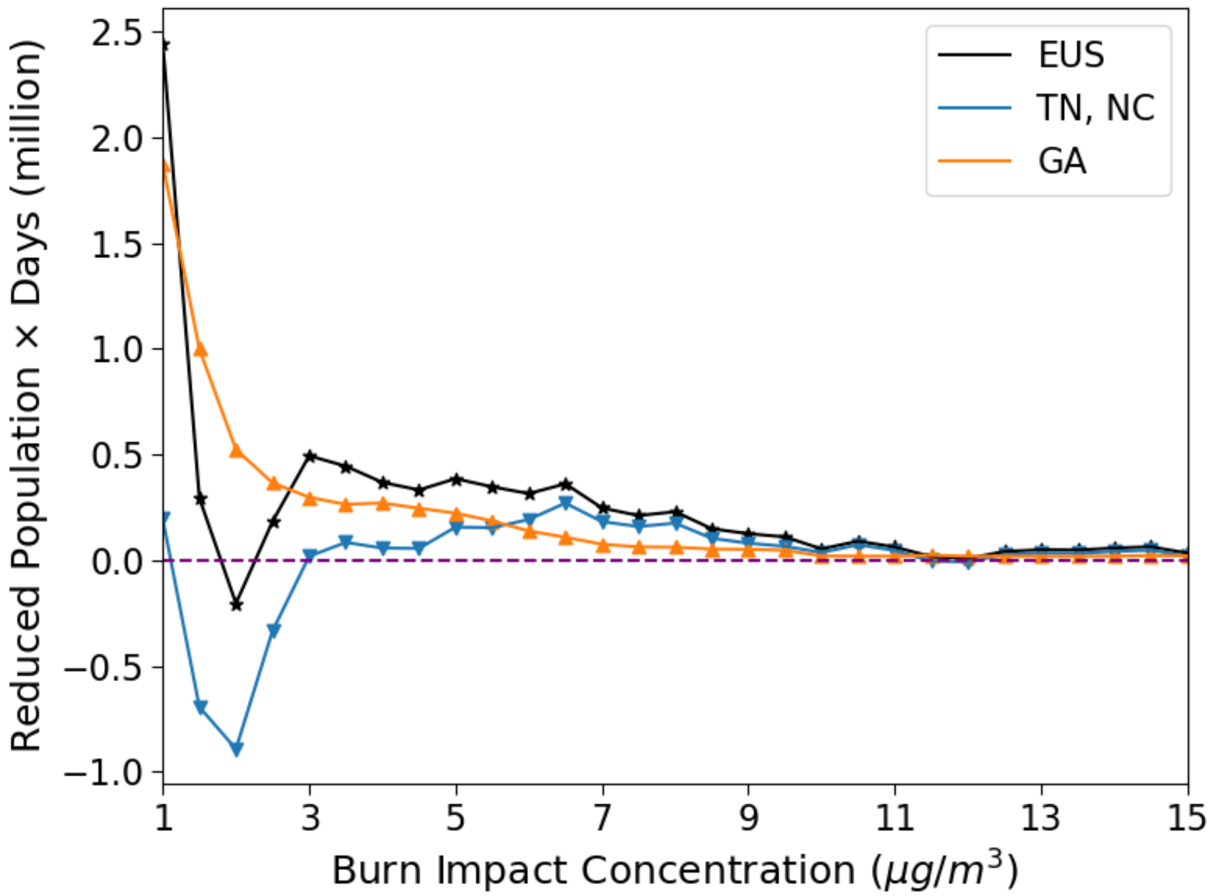

Figure S14. The prescribed fire prevented person-days (unit: million person-day) under different spatial ranges for a certain burn impact concentration threshold. The dashed line shows zero. The sum of prescribed fire and post-prescribed burn wildfire has lower person-days than wildfire when the line is above the dashed line. EUS: Eastern U.S. (the entire study domain; same as the black line in Figure 5); TN-NC: Tennessee and North Carolina; GA: Georgia.

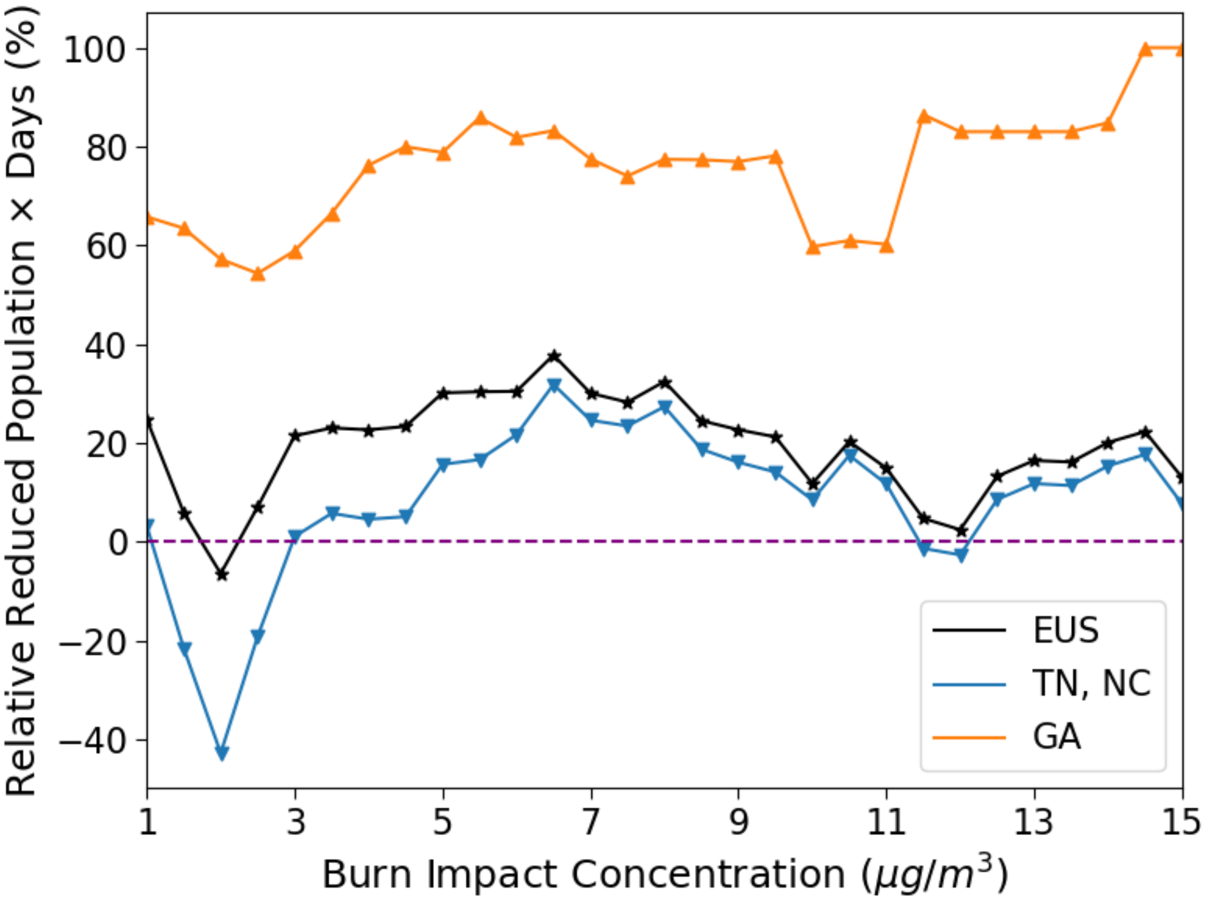

Figure S15. The relative prescribed fire prevented person-days compared to wildfire person-time under different spatial ranges for a certain burn impact concentration threshold. The dashed line shows zero. The relative reduced person days is calculated by:$(PD_{WF} - PD_{Rx} - PD_{post-Rx\,WF})/PD_{WF}$, where PD is person-days. EUS: Eastern U.S. (the entire study domain); TN-NC: Tennessee and North Carolina; GA: Georgia.

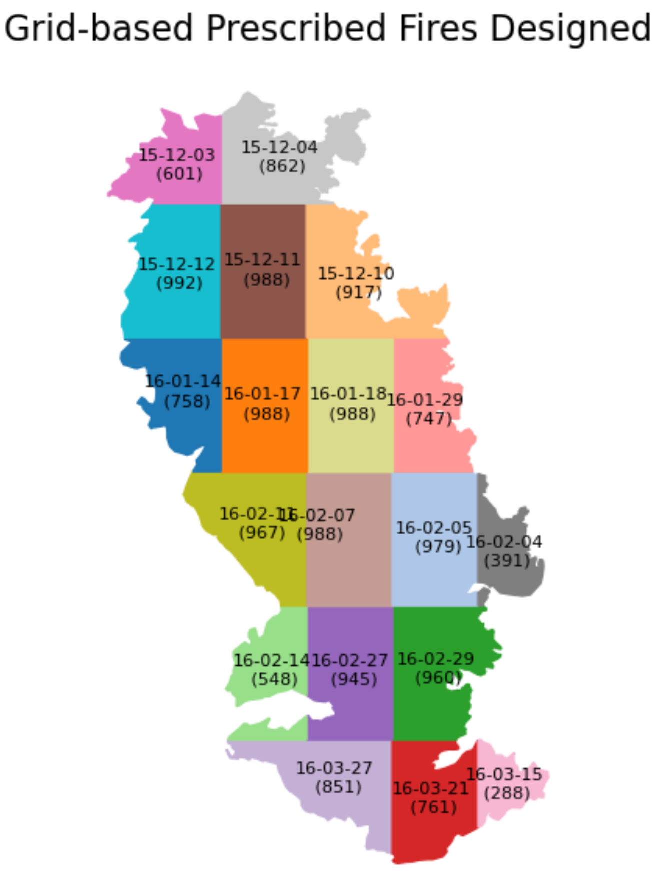

Figure S16. Grid-based prescribed fire boundary design. The prescribed fire boundary is the intersection between a rectangular grid and the Gatlinburg wildfire boundary. The burn dates are the same as the prescribed fire design with fire break considerations. However, the burned area for each date is different due to the different fire boundaries.

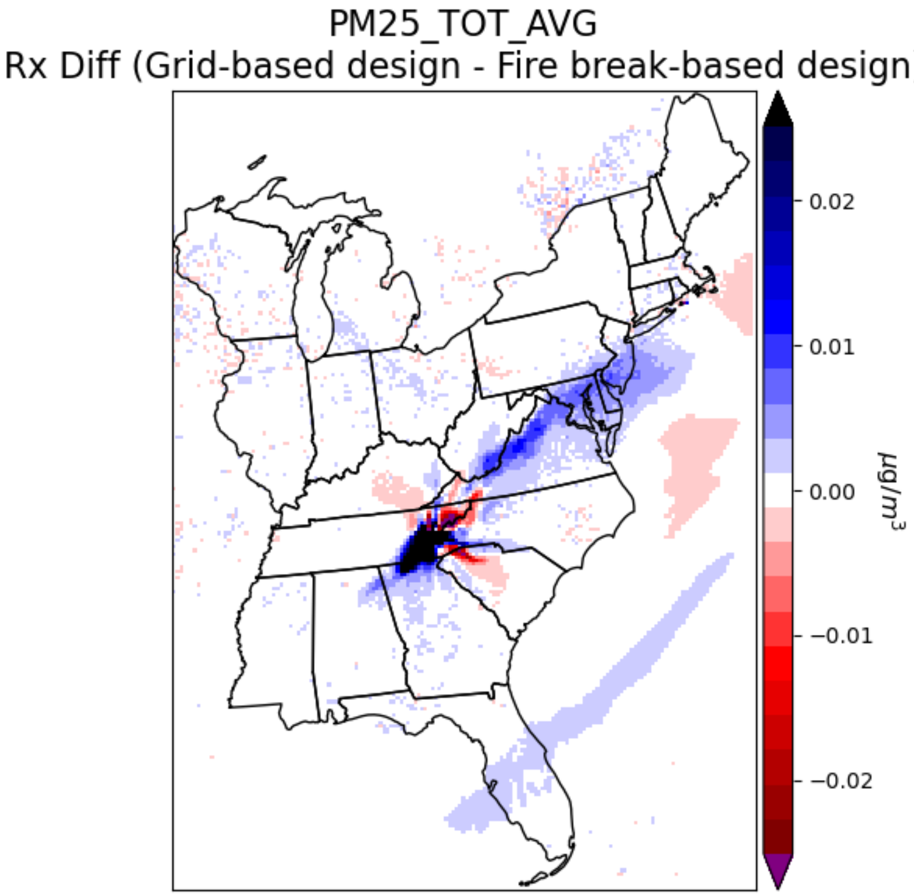

Figure S17. The mean difference of daily average $PM_{2.5}$ between grid-based design and fire break-based design during the prescribed fire burn dates. The blue tones show places where the grid-based design leads to higher $PM_{2.5}$ concentrations than the fire break-based design. The red tones show places where the grid-based design leads to lower $PM_{2.5}$ concentrations than the fire break-based design.

Table S4. Designed prescribed burns region area, the non-burnable (developed/barren land) area, and the effective burned area in FCCS and NLCD datasets (unit: acres).

Burn #	Rx burn date	Region Area	FCCS V2 Fuel Load Map		NLCD Map	
			Developed/ barren land area	Effective burned area	Developed/ barren land area	Effective burned area
1	2015-12-03	637	0	637	137	500
2	2015-12-04	459	0	459	86	373
3	2015-12-10	863	0	863	296	567
4	2015-12-11	934	0	934	396	538
5	2015-12-12	977	54	922	204	773
6	2016-01-14	1233	193	1040	753	480
7	2016-01-17	910	0	910	52	858
8	2016-01-18	817	0	817	40	776
9	2016-01-29	578	0	578	26	552
10	2016-02-04	763	0	763	20	743
11	2016-02-05	466	0	466	19	447
12	2016-02-07	964	0	964	15	949
13	2016-02-11	894	0	894	1	893
14	2016-02-14	922	0	922	0	922
15	2016-02-27	915	0	915	19	896
16	2016-02-29	487	0	487	0	487
17	2016-03-15	749	0	749	19	731
18	2016-03-21	960	0	960	18	943
19	2016-03-27	995	0	995	0	995

Table S5. Daily averaged $PM_{2.5}$, MDA8-O_3, and 1-hr max NO_2 performance during the study, i.e., November 25th, 2016 to December 1st, 2016 in Alabama, Georgia, Kentucky, Virginia, North Carolina, South Carolina, and Tennessee., which covers the Gatlinburg wildfire and the following two days.

	**Daily averaged $PM_{2.5}$**	**MDA8-O_3**	**1-hr max NO_2**
MB	-2.42	-1.22	-2.63
ME	3.14	3.78	6.27
RMSE	4.47	4.75	8.28
CRMSE	3.76	4.59	7.85
NMB	-29.62	-3.74	-14.68
NME	38.41	11.51	34.95
MNB	-22.94	-2.07	15.32
MNE	36.65	11.60	54.49
FB	-34.53	-3.17	-8.11
FE	44.79	11.90	43.34
IOA	0.63	0.70	0.78
Pearson R	0.49	0.55	0.66

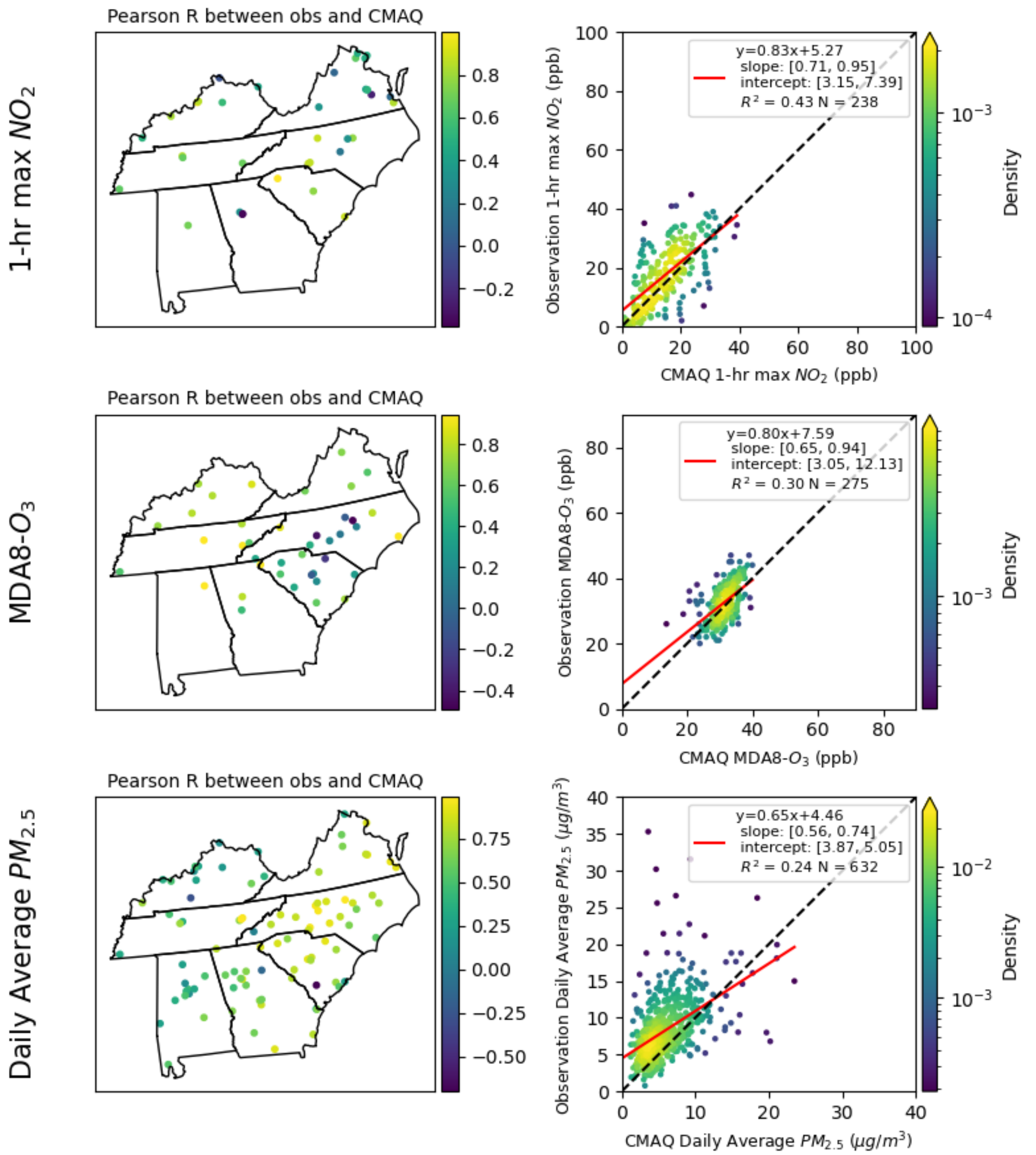

Figure S18. Model evaluation by comparing simulations to observations for daily average $PM_{2.5}$, MDA8-O_3, and 1-hr max NO_2 (the wildfire burn dates and the following two days, Nov 25th, 2016 to Dec 1st, 2016) in Alabama, Georgia, Kentucky, Virginia, North Carolina, South Carolina, and Tennessee. The spatial plots on the left show the Pearson correlation coefficient (also known as R) value between simulation and observation for each monitor in the study domain. The density scatterplots on the right show the relationship between all observations and simulations. The black dashed line is the unity (1:1) slope line. The red line shows the linear relationship between simulation and observations. The R^2 performance and 95% confidential interval of

slopes and intercepts of the regression line are indicated. N shows the total number of data points in the linear regression.

Table S6. Model performance for daily averaged $PM_{2.5}$, MDA8-O_3, and 1-hr max NO_2 in Scenario 2 during the designed counterfactual prescribed burn days and the two days following each burn (for a total of 45 days between December 3rd 2015 and March 29th, 2016 as listed in Table S8), in the focused states (shown by the red boundaries in Figure 1).

	**Daily averaged $PM_{2.5}$**	**MDA8-O_3**	**1-hr max NO_2**
MB	0.10	-2.37	-4.95
ME	3.05	5.13	8.57
RMSE	4.61	6.57	11.46
CRMSE	4.61	6.12	10.34
NMB	1.18	-6.59	-22.68
NME	36.32	14.26	39.3
MNB	18.34	0.01	-8.46
MNE	49.14	18.53	45.96
FB	-2.61	-4.65	-26
FE	36.81	15.93	49.39
IOA	0.80	0.84	0.77
Pearson R	0.66	0.79	0.63

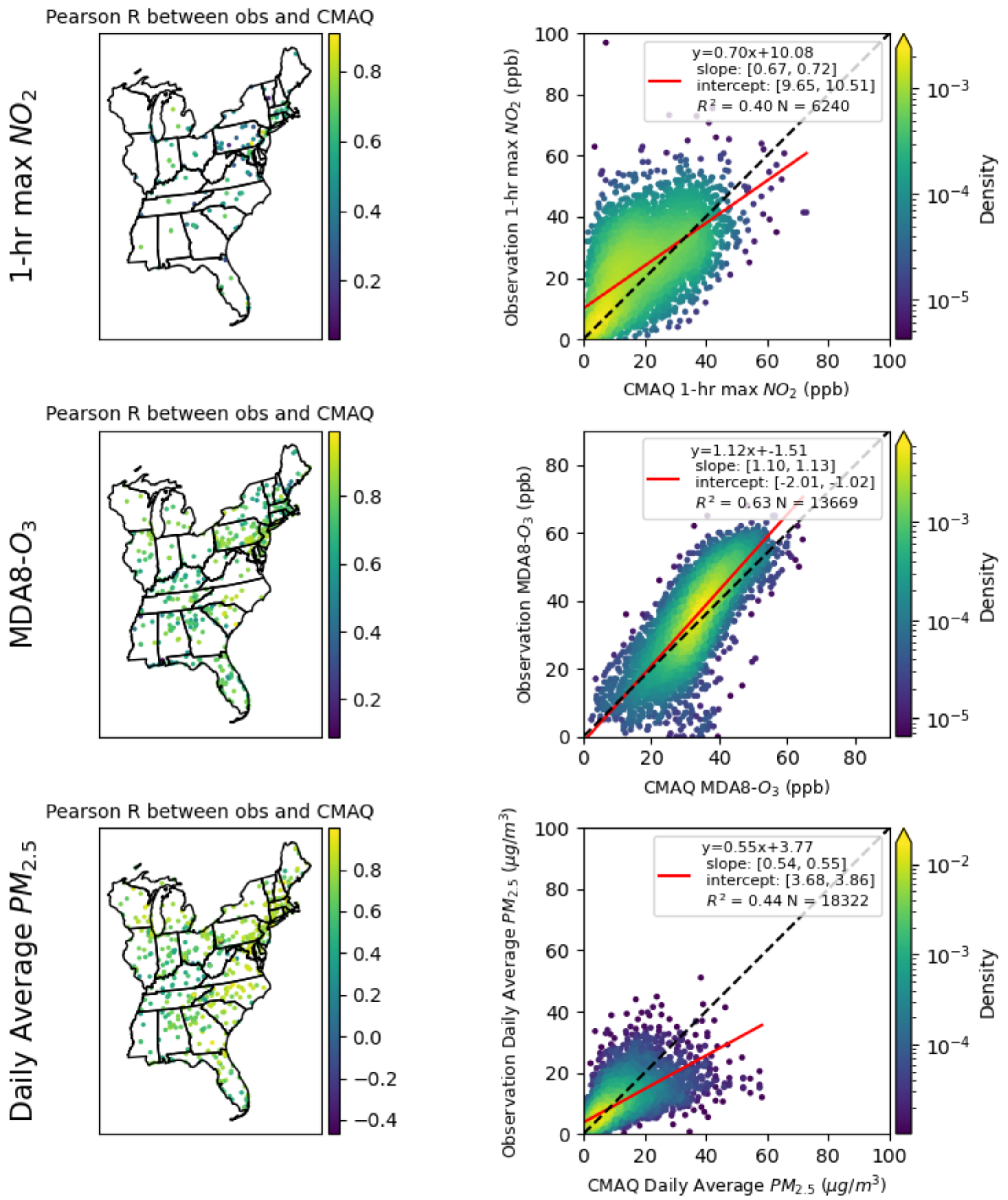

Figure S19. Model evaluation by comparing Scenario 2 simulation results to observations for daily average $PM_{2.5}$, MDA8-O_3, and 1-hr max NO_2 on counterfactual prescribed burn days and the following two days. The spatial plots on the left show the Pearson correlation coefficient value at each monitor in the study domain. The density scatterplots on the right show the relationship between observations and simulation results. The black dashed line is the unity (1:1) slope line. The red line is the linear regression line whose equation is shown along with the 95% confidence interval for the slope and intercept. R^2 is the coefficient of determination and N is the number of data points in the linear regression.

Table S7. Model performance for daily averaged $PM_{2.5}$, MDA8-O_3, and 1-hr max NO_2 in Scenario 2 during the Gatlinburg wildfire period and the following two days (for a total of 7 days between November 25th, 2016 and December 1st, 2016) in the focused states (shown by the red boundaries in Figure 1).

	**Daily averaged $PM_{2.5}$**	**MDA8-O_3**	**1-hr max NO_2**
MB	-1.80	0.68	-1.95
ME	3.23	4.10	6.43
RMSE	4.62	5.39	8.61
CRMSE	4.25	5.35	8.38
NMB	-22.10	2.47	-10.38
NME	39.72	15.00	34.19
MNB	-9.65	12.77	10.57
MNE	42.85	22.82	46.80
FB	-25.24	6.34	-6.53
FE	45.64	17.30	41.32
IOA	0.71	0.85	0.80
Pearson R	0.56	0.80	0.66

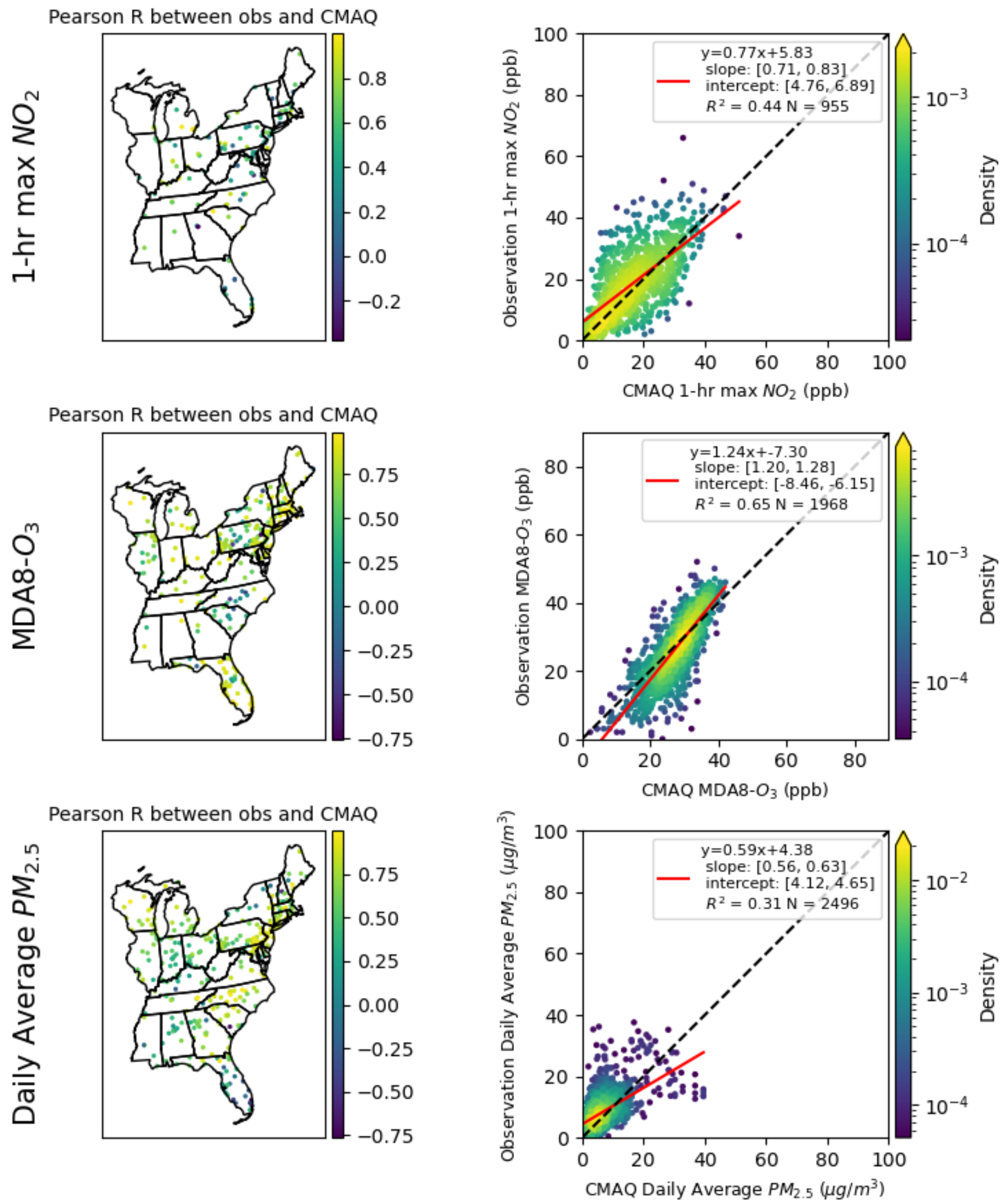

Figure S20. Model evaluation by comparing Scenario 2 simulation results to observations for daily average $PM_{2.5}$, MDA8-O_3, and 1-hr max NO_2 on the wildfire days and the following two days. The spatial plots on the left show the Pearson correlation coefficient value at each monitor in the study domain. The density scatterplots on the right show the relationship between observations and simulation results. The black dashed line is the unity (1:1) slope line. The red line is the linear regression line whose equation is shown along with the 95% confidence interval for the slope and intercept. R^2 is the coefficient of determination and N is the number of data points in the linear regression.

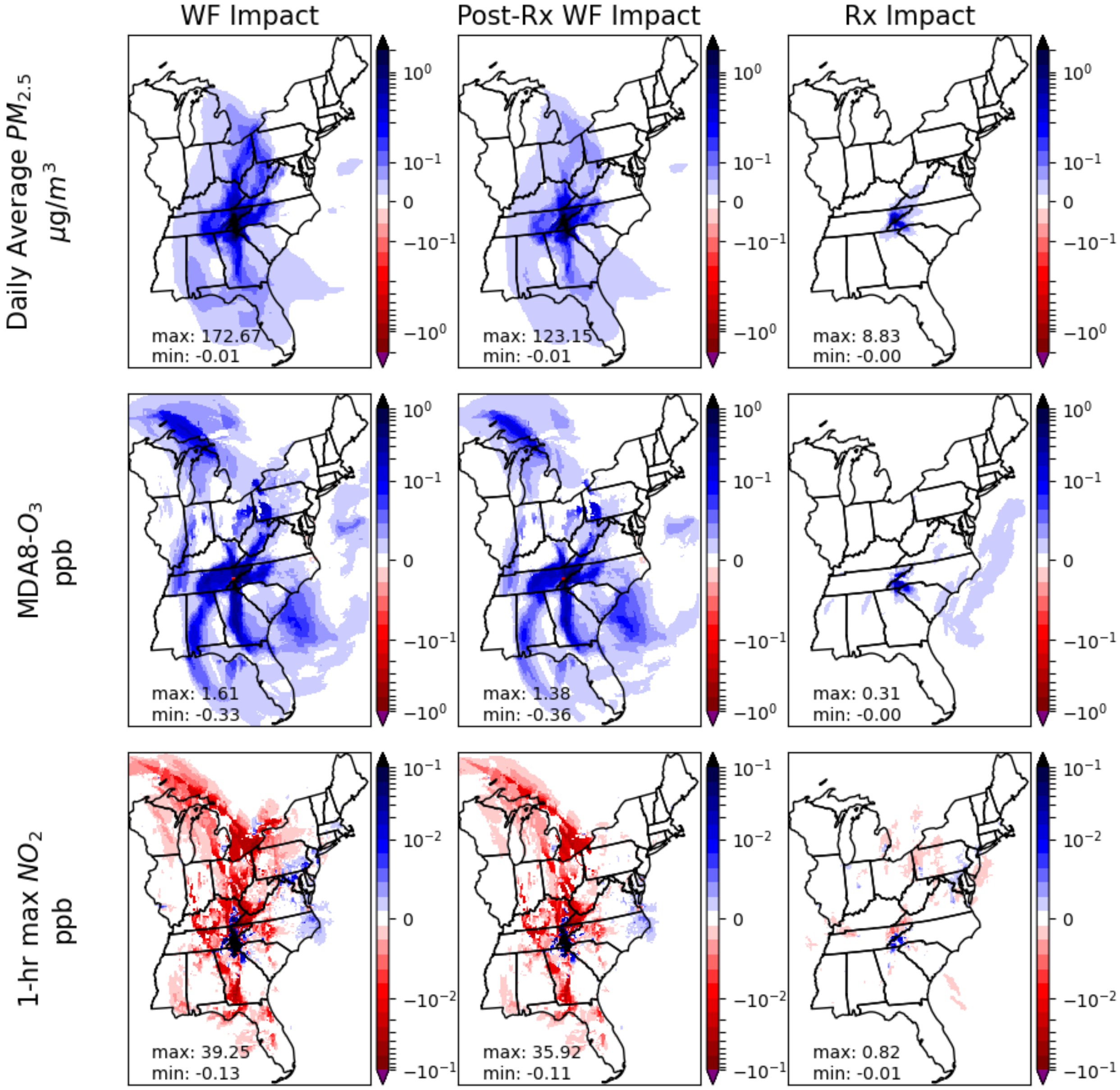

Figure S21. Mean daily average $PM_{2.5}$, MDA8-O_3, and 1-hr max NO_2 of smoke impacts during the wildfire, post-prescribed burn wildfire, and prescribed burns periods. The smoke impacts are calculated by subtracting the baseline scenario concentrations from fire cases. The impacts are plotted using the same symmetrical log scale for all three types of fires.

Table S8. Burn dates, CMAQ simulation and study focused time periods for different fire cases.

Fire cases name	Burn dates	CMAQ simulation periods	Study focused periods
WF	2016-11-25 to 2016-11-29	2016-11-23 to 2016-12-04	2016-11-25 to 2016-12-01
Post-Rx WF	2016-11-25 to 2016-11-29	2016-11-23 to 2016-12-04	2016-11-25 to 2016-12-01
Rx	2015-12-03<br>2015-12-04<br>2015-12-10<br>2015-12-11<br>2015-12-12<br>2016-01-14<br>2016-01-17<br>2016-01-18<br>2016-01-29<br>2016-02-04<br>2016-02-05<br>2016-02-07<br>2016-02-11<br>2016-02-14<br>2016-02-27<br>2016-02-29<br>2016-03-15<br>2016-03-21<br>2016-03-27	2015-12-01 to 2015-12-16<br>2016-01-12 to 2016-04-01	2015-12-03 to 2015-12-06,<br>2015-12-10 to 2015-12-14,<br>2016-01-14 to 2016-01-20,<br>2016-01-29 to 2016-01-31,<br>2016-02-04 to 2016-02-09,<br>2016-02-11 to 2016-02-16,<br>2016-02-27 to 2016-03-02,<br>2016-03-15 to 2016-03-17,<br>2016-03-21 to 2016-03-23,<br>2016-03-27 to 2016-03-29

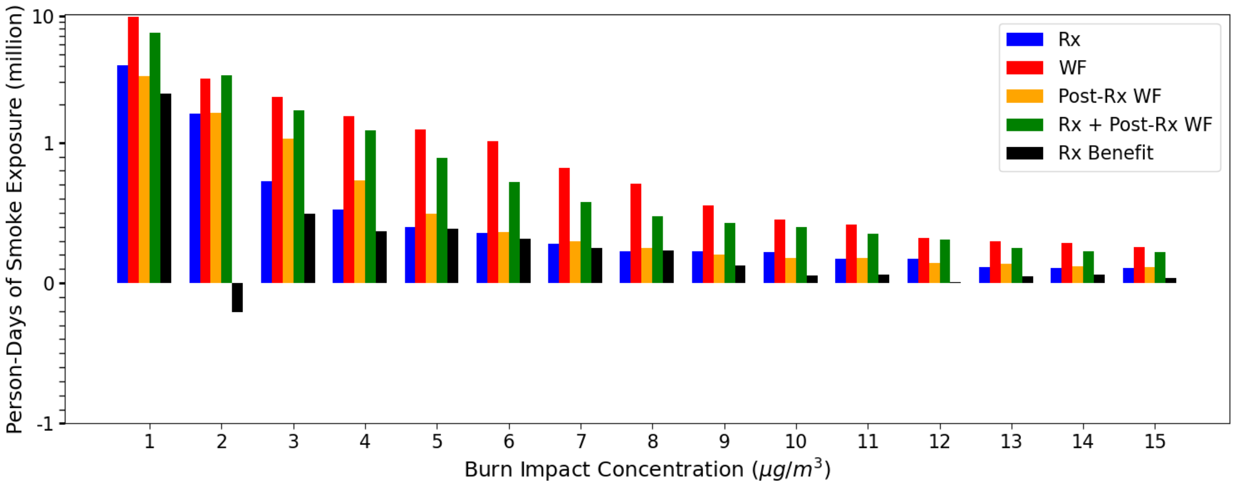

Figure S22. The person-days under prescribed fire (Rx: blue), wildfire (WF: red), and post-prescribed burn wildfire (Post-Rx WF: orange) cases, for burn impact concentration threshold from 1 to 15 μg/m^3 at 1 μg/m^3 intervals. The y-axis is in symmetrical log scale. The green bars show the sum of person-days from Rx and Post-Rx WF. The black bars show the person-days prevented by prescribed fires, calculated as the difference between person-days of wildfire exposure and the combined person-days of prescribed fire and post-prescribed burn wildfire exposure.

Text S2. Scenario 3*

Scenario 3* design:

We designed a new scenario (Scenario 3*) to address the situation where prescribed burn treatment does not cover all of the Gatlinburg wildfire area. We split the burned area in two approximately equal parts and assumed that prescribed burn treatment was applied to only one of them (the part demarcated with the blue boundary in Figure S23). We chose the blue boundary part rather than the red boundary part in Figure S23 for prescribed burn treatment because the wildfire originated in the blue boundary part and the red boundary part is closer to the Gatlinburg urban area (Figure S4) which could make treatment more challenging. We then calculated the wildland fire emissions for Scenario 3* using the BlueSky pipeline as follows.

Prescribed burns (Rx*): We selected 10 prescribed burns (covering the blue boundary part in Figure S23) from the 19 prescribed burns in Scenario 3 and assumed these burns were conducted before the wildfire occurrence on the same days as in Scenario 3. The emissions for these 10 prescribed burns were already calculated as part of Scenario 3.

Post-prescribed burn wildfire (Post-Rx WF*): For the blue boundary part (the part with prescribed burn treatment), we applied the same method to calculate the post-prescribed burn wildfire emission as in Scenario 3. Specifically, we subtracted the fuel consumed in prescribed burns from the fuel load before calculating the post-prescribed burn wildfire emissions with the BlueSky pipeline. We used 20% canopy consumption for this part as we did for the post-prescribed burn wildfire in Scenario 3. For the red boundary part (the part without prescribed burn treatment), we applied the same settings as the wildfire case in Scenario 3. In other words, we did not subtract any fuel load since there was no prescribed burn treatment in this part and we used 50% canopy consumption.

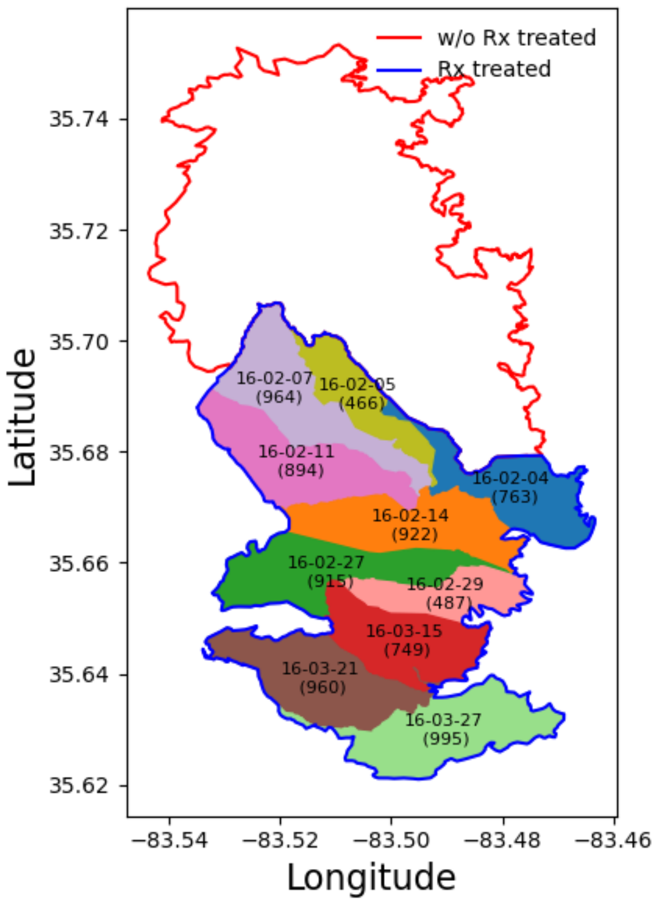

Figure S23. Blue line indicates the part of the Gatlinburg wildfire area treated with prescribed burns in Scenario 3*. Each prescribed burn's boundary is filled with a different color. The first line of text in each boundary indicates the burn date (year-month-day in YYMMDD format), and the second line indicates the burned area (acres). Red line shows the boundary of the part without prescribed burn treatment.

Emissions:

The total emissions of NO_x, $PM_{2.5}$, and VOC for Scenario 3* were slightly higher than Scenario 3, specifically by 8.0, 29.7, and 15.3 tons, respectively (Table S9). In Scenario 3, Rx contributed more emissions than post-Rx WF. However, in Scenario 3*, post-Rx WF* became the primary source of emissions.

Table S9. Wildland fire emissions (unit: metric tons) under different scenarios.

**Scenario Name**	**Fire Type**	**NO_x**	**$PM_{2.5}$**	**VOC**
Scenario 2	WF	736.3	5609.4	6307.6
	Total:	736.3	5609.4	6307.6
Scenario 3	Rx	359.9	2914.3	3322.1
	Post-Rx WF	301.0	2363.0	2697.8

Total			660.9	5277.3	6019.9
Scenario 3*	Rx* (red boundary part in Figure S23)		189.0	1592.2	1829.6
	Post-Rx WF*	(red boundary part)	188.8	1505.9	1720.9
		(blue boundary part)	291.1	2208.9	2484.7
Total			668.9	5307.0	6035.2

Smoke Person-days Analysis:

To understand the air quality tradeoffs between Scenario 2 and Scenario 3*, we used the same method to calculate the smoke person-days of $PM_{2.5}$ under different exposure levels (Figure S24). Similar to Scenario 3, prescribed burning has positive benefits of preventing population exposures for low ($PM_{2.5} \leqslant 1$ μg/m^3) and high levels ($PM_{2.5} \geqslant 3$ μg/m^3) of $PM_{2.5}$. However, there are some disbenefits around 2 μg/m^3.

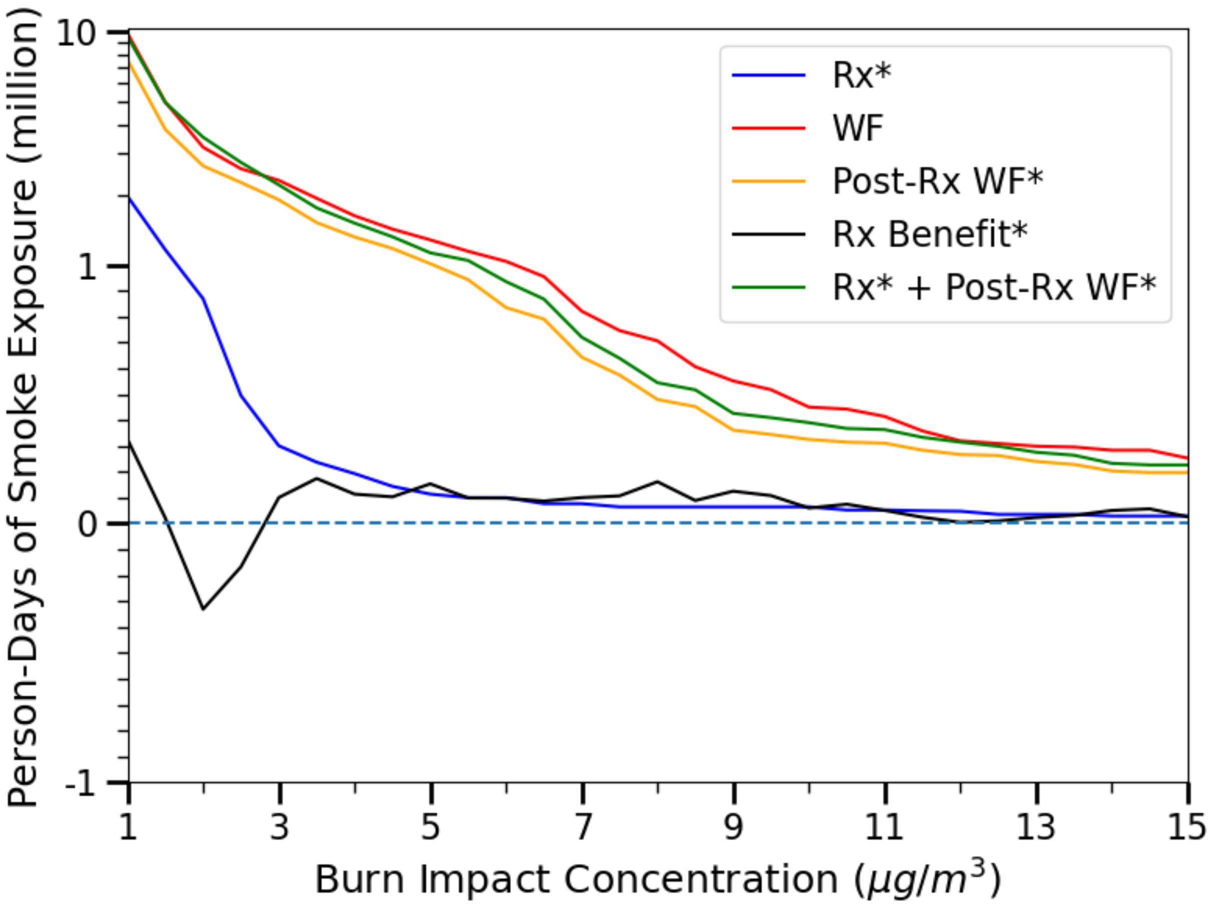

Figure S24. The person-days under Gatlinburg wildfire (WF) and prescribed fire (Rx*) and post-prescribed burn wildfire (Post-Rx WF*) cases of Scenario 3* for specific burn impact concentration thresholds, represented by red, blue, and orange lines, respectively. The green line shows the sum of person-days for Scenario 3*. The black line shows the person-days prevented by prescribed burns Rx*, calculated as the difference in person-days of exposure between Gatlinburg wildfire and Scenario 3*. The dashed line represents zero person-days.

Disclaimer

The contents of this article do not necessarily reflect the views of HEI or its sponsors, nor do they necessarily reflect the views and policies of the EPA or motor vehicle and engine manufacturers. The findings and conclusions in this study are those of the authors and do not necessarily represent the official position of the Centers for Disease Control and Prevention (CDC).

Reference:

1. NOAA. Gridded Meteorological Data Archives NAM 12km. Available online: https://www.ready.noaa.gov/archives.php (accessed on 2023, May 21).
2. Ottmar, R.D.; Sandberg, D.V.; Riccardi, C.L.; Prichard, S.J. An overview of the fuel characteristic classification system—quantifying, classifying, and creating fuelbeds for resource planning. *Canadian Journal of Forest Research* **2007**, *37*, 2383-2393.
3. Deeming, J.E. *National fire-danger rating system*; Rocky Mountain Forest and Range Experiment Station, Forest Service, US ...: 1972; Volume 84.
4. Ottmar, R.D.; Burns, M.F.; Hall, J.N.; Hanson, A.D. CONSUME: users guide. *Gen. Tech. Rep. PNW-GTR-304. Portland, OR: US Department of Agriculture, Forest Service, Pacific Northwest Research Station.* **1993**, *304*.
5. Prichard, S.J.; O'Neill, S.M.; Eagle, P.; Andreu, A.G.; Drye, B.; Dubowy, J.; Urbanski, S.; Strand, T.M. Wildland fire emission factors in North America: synthesis of existing data, measurement needs and management applications. *International Journal of Wildland Fire* **2020**, *29*, 132-147.
6. Briggs, G.A. Plume rise and buoyancy effects. *Atmospheric science and power production* **1984**, *327*, 366.
7. Emery, C.; Liu, Z.; Russell, A.G.; Odman, M.T.; Yarwood, G.; Kumar, N. Recommendations on statistics and benchmarks to assess photochemical model performance. *Journal of the Air & Waste Management Association* **2017**, *67*, 582-598.
8. Lelieveld, J.; Barlas, C.; Giannadaki, D.; Pozzer, A. Model calculated global, regional and megacity premature mortality due to air pollution. *Atmospheric Chemistry and Physics* **2013**, *13*, 7023-7037.
9. Roth, G. Global Burden of Disease Collaborative Network. Global Burden of Disease Study 2017 (GBD 2017) Results. Seattle, United States: Institute for Health Metrics and Evaluation (IHME), 2018. *The Lancet* **2018**, *392*, 1736-1788.
10. Chen, G.; Guo, Y.; Yue, X.; Tong, S.; Gasparrini, A.; Bell, M.L.; Armstrong, B.; Schwartz, J.; Jaakkola, J.J.; Zanobetti, A. Mortality risk attributable to wildfire-related PM2· 5 pollution: a global time series study in 749 locations. *The Lancet Planetary Health* **2021**, *5*, e579-e587.
11. Bell, M.L.; Dominici, F.; Samet, J.M. A meta-analysis of time-series studies of ozone and mortality with comparison to the national morbidity, mortality, and air pollution study. *Epidemiology* **2005**, *16*, 436-445.
12. Wang, M.; Li, H.; Huang, S.; Qian, Y.; Steenland, K.; Xie, Y.; Papatheodorou, S.; Shi, L. Short-term exposure to nitrogen dioxide and mortality: a systematic review and meta-analysis. *Environmental research* **2021**, *202*, 111766.
13. EPA. Environmental Benefits Mapping and Analysis Program–Community Edition User's. *Manual and Appendices* **2018**.
14. Pan, S.; Gan, L.; Jung, J.; Yu, W.; Roy, A.; Diao, L.; Jeon, W.; Souri, A.H.; Gao, H.O.; Choi, Y. Quantifying the premature mortality and economic loss from wildfire-induced PM2. 5 in the contiguous US. *Science of The Total Environment* **2023**, *875*, 162614.
15. Pan, S.; Yu, W.; Fulton, L.M.; Jung, J.; Choi, Y.; Gao, H.O. Impacts of the large-scale use of passenger electric vehicles on public health in 30 US. metropolitan areas. *Renewable and Sustainable Energy Reviews* **2023**, *173*, 113100.

16. Amann, M. *Health risks of ozone from long-range transboundary air pollution*; WHO Regional Office Europe: 2008.
17. Yang, J.; Zhao, Y.; Cao, J.; Nielsen, C.P. Co-benefits of carbon and pollution control policies on air quality and health till 2030 in China. *Environment international* **2021**, *152*, 106482.
